# Ultrasound-enhanced exosome secretion and antibody-free SERS profiling for Alzheimer’s disease via iPSC-derived cortical organoids

**DOI:** 10.1126/sciadv.aed3930

**Published:** 2026-09-23

**Authors:** Jinhee Yoo, Youngjun Kim, Won Jong Yu, Seongmin Kim, Yongjae Jo, Hanjun Cho, Hyunsu Park, Luke P. Lee, Jong-Chan Park, Inki Kim, Byullee Park

**Affiliations:** ^1^ Department of Biophysics, Institute of Quantum Biophysics, Sungkyunkwan University, Suwon 16419, Republic of Korea.; ^2^ Department of Metabiohealth, Sungkyunkwan University, Suwon 16419, Republic of Korea.; ^3^ Department of Biopharmaceutical Convergence, Sungkyunkwan University, Suwon 16419, Republic of Korea.; ^4^ Department of Medicine, Brigham and Women’s Hospital, Harvard Medical School, Harvard University, Boston, MA 02115, USA.; ^5^ Department of Intelligent Precision Healthcare Convergence, Sungkyunkwan University, Suwon 16419, Republic of Korea.

## Abstract

Exosomes offer a promising vehicle for noninvasive diagnosis of neurodegenerative diseases by carrying multiple biomarkers across the blood-brain barrier. However, traditional exosome analysis often misses subtle genotype-specific variations because of weak signals from a few exosomes, reliance on markers, and limited understanding of disease signatures. Here, we present a sound way to understand Alzheimer’s disease through label-free surface-enhanced Raman scattering (SERS) profiling of cortical organoid-derived exosomes. Initially, we enhanced exosomal SERS signal levels by optimizing ultrasound parameters to increase exosome secretion from cortical organoids. Using this strategy, we distinguished apolipoprotein E genotype–dependent differences in intact exosomal fingerprints. Transcriptome analysis confirmed that ultrasound stimulation increased exosome secretion and up-regulated exosome-related genes functionally linked to Alzheimer’s pathology. Ultrasound-based stimulation and label-free exosome profiling reveal genotype-specific pathologies, providing a precision-diagnostic framework for Alzheimer’s disease.

## INTRODUCTION

Exosomes are nanoscale extracellular vesicles secreted by virtually all cell types, containing proteins, lipids, and nucleic acids, that reflect the physiological and pathological states of their cells of origin (*1*, *2*). Owing to their stability in biofluids and ability to cross biological barriers, exosomes have emerged as promising biomarkers for minimally invasive diagnostics across diverse diseases, including cancer, cardiovascular conditions, and neurodegenerative disorders (*3*, *4*). In the context of Alzheimer’s disease (AD), exosomes can carry hallmark molecules such as amyloid-β (Aβ) and provide genetic information such as apolipoprotein E (APOE) genotype, and cross the blood-brain barrier, both of which are clinically relevant for disease risk stratification (*5*, *6*). However, to unlock the diagnostic potential of exosomes, it is essential to develop analytical platforms capable of accurately and sensitively detecting their molecular contents.

Various analytical techniques have been developed to characterize exosomes, each with its unique strengths and limitations. Nanoparticle tracking analysis (NTA) is widely used to estimate size and concentration but requires high particle concentrations (>10^7^ particles/ml) (*7*, *8*). Electrochemical biosensors offer simple, low-cost detection, but are limited by their relatively low sensitivity and specificity (*9*). Plasmonic sensing technologies, including surface plasmon resonance (SPR) and surface-enhanced Raman scattering (SERS), provide sensitive molecular profiling capabilities (*10*–*14*). SPR enables the real-time monitoring of specific surface interactions, whereas SERS amplifies molecular fingerprints with ultrahigh sensitivity, even at the single-vesicle level. To further enhance signal detection, these methods have used labeling (*15*) or preprocessing, such as exosome lysis or enzymatic treatment (*10*, *11*), but they often lead to sample loss and limit the accuracy of complex signal analysis.

Recent advancements in SERS have introduced antibody-free substrates and intact exosome profiling methods that streamline workflows and preserve biomarker integrity while also enabling multiplexed analysis (*16*, *17*). They classified cancer types by analyzing the SERS spectral patterns from cancer-derived intact exosomes, leveraging diverse molecular signatures rather than relying on predefined biomarkers. However, detecting subtle genetic differences, such as APOE genotypes, remains a critical challenge because of the limited sensitivity of exosomal surface signals. Addressing these genotype-specific variations requires multiplexed analysis of diverse exosomal biomarkers, higher vesicle yields, and improved signal amplification strategies.

**Fig. 1.**
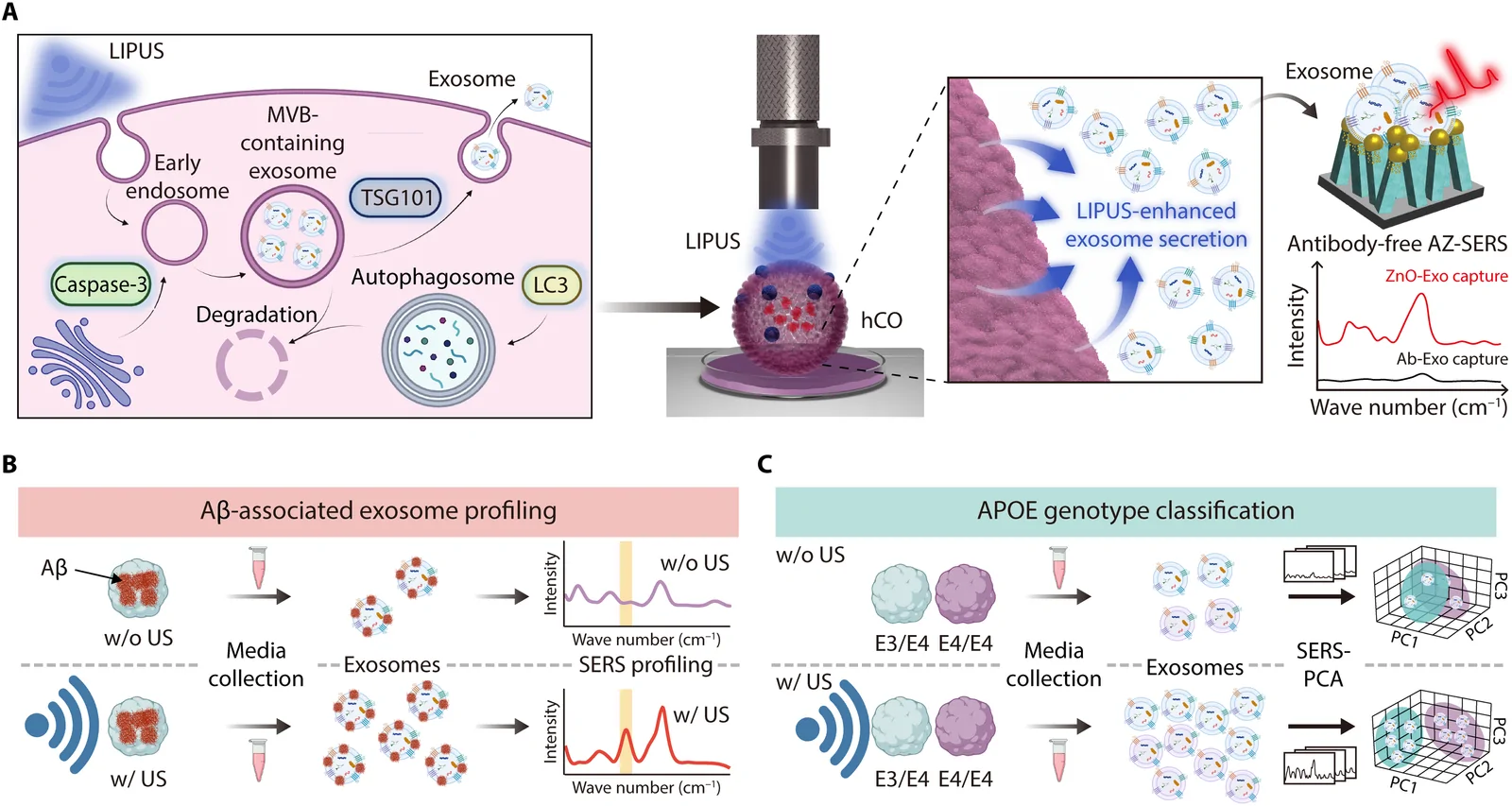
Ultrasound-enhanced exosome secretion and antibody-free SERS profiling. (**A**) Conceptual workflow showing LIPUS-enhanced exosome secretion from hCOs, ultrasound-responsive exosome release pathways, and antibody-free AZ-SERS profiling of intact exosomes. (**B**) Application to Aβ-associated exosome profiling with or without LIPUS stimulation. (**C**) Application to APOE genotype classification using SERS-PCA analysis of hCO-derived exosomes with or without LIPUS stimulation. w/o, without; w/, with; US, ultrasound; PC, principal component; MVB, multivesicular body. Created in BioRender. Kim, Y. (2026) https://BioRender.com/f9dq32u.

In this study, we developed a synergistic system that combines ultrasound-induced exosome enhancement with antibody-free SERS detection (US-SERS system) to capture APOE genotype–dependent pathological differences (Fig. 1A). We optimized the ultrasound parameters with real-time SERS monitoring, which revealed that low-intensity pulsed ultrasound (LIPUS) increased the exosome-associated SERS signal by approximately 2.3-fold, supporting increased exosome secretion (*18*–*24*). We found that LIPUS activated the endosomal sorting complexes required for transport (ESCRT) and apoptosis, thereby promoting endosome formation and exosome release. Excessive intensity triggered autophagy, which suppressed exosome secretion. To enable antibody-free SERS detection, we fabricated a gold-coated zinc oxide nanowire–based SERS (AZ-SERS) substrate that captures exosomes via electrostatic interactions, thereby minimizing the gap to the SERS-active surface. Under the same calibration-based statistical limit of detection (LOD) analysis framework, AZ-SERS yielded an extrapolated LOD estimate of 69 particles/ml, compared with 105 particles/ml for antibody-functionalized substrates. AZ-SERS also enabled reliable quantification at 10^4^ particles/ml, below the practical NTA measurement range (>10^7^ particles/ml).

In addition, we observed a reproducible enhancement of a SERS peak at 1362 cm^−1^ that responded sensitively to the presence and increased secretion of Aβ-containing exosomes, indicating that exosome-associated molecular changes correlated with Aβ can be detected within intact exosomes (Fig. 1B). We further clarified the pathological relevance of the APOE genotype (E3 versus E4) in multiplexed signals by AZ-SERS–integrated principal components analysis (PCA), increasing the silhouette scores from 0.37 to 0.50 (Fig. 1C). Transcriptomic analysis revealed that LIPUS stimulation not only increased exosome secretion, but also up-regulated genes involved in protein synthesis, neurotransmitter metabolism, and stress responses. Notably, several exosome-associated genes were directly connected to APOE and amyloid precursor protein (APP), linking the ultrasound–exosome pathway to AD pathology. By using human cortical brain organoids (hCOs), our study provides a controlled, brain-relevant system that minimizes patient-derived variability, enables genotype-specific comparisons, and allows the mechanistic interrogation of ultrasound-exosome pathways. To the best of our knowledge, this is the first study to combine ultrasound-induced exosome enhancement with antibody-free SERS detection to enable APOE genotype clustering based on intact exosomal signals. Given that ultrasound is a scalable stimulus from cells to whole organisms, this strategy holds promise for enabling genotype-level diagnostics across diverse biological systems using exosomal biomarkers, thereby facilitating clinical translation.

## RESULTS

### Antibody-free SERS substrate for sensitive exosome capture

We engineered an AZ-SERS substrate that was optimized for direct exosome capture (fig. S1). A ZnO seed layer was prepared by spin coating and baking, as its quality critically determines the nanowire morphology, crystallinity, and subsequent SERS sensitivity (*25*). ZnO nanowires were then grown on the seed layer via hydrothermal synthesis, thereby forming high-density, well-aligned nanowire structures at low temperatures, facilitating efficient exosome capture between the nanowires (*26*). Last, a 100-nm gold film was deposited on the substrates by thermal evaporation to generate plasmonic hotspots.

**Fig. 2.**
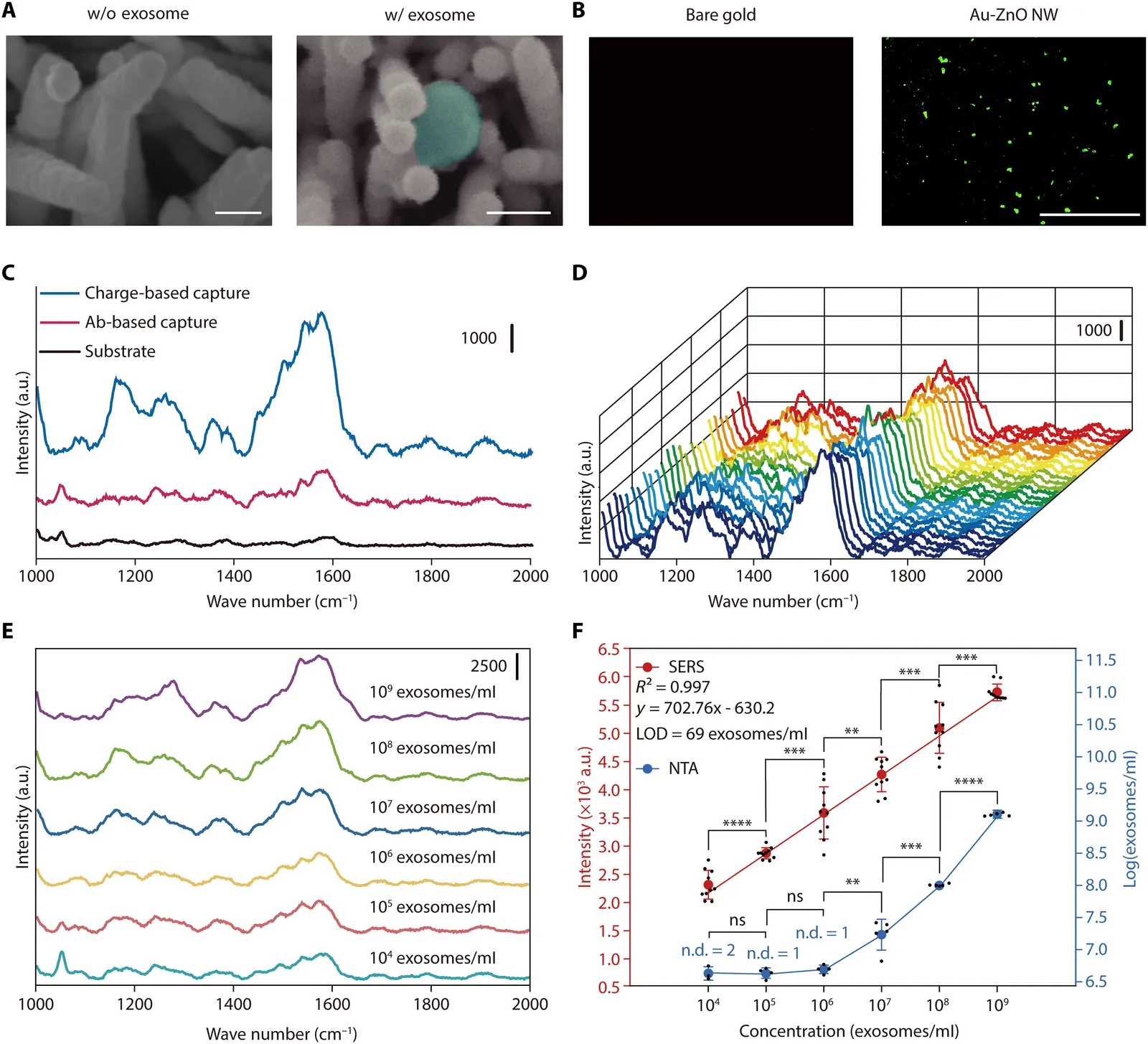
Antibody-free AZ-SERS substrate for exosome capture and quantitative SERS analysis. (**A**) SEM images of the AZ-SERS substrate without and with exosome attachment. Scale bars, 100 nm. (**B**) Fluorescence imaging of exosome adhesion on bare gold and Au-ZnO nanowire surfaces. Scale bar, 200 μm. (**C**) Comparison of SERS intensity between antibody-free and antibody-functionalized substrates. (**D**) Uniformity of SERS signals across the substrate. (**E**) SERS spectra of exosomes depending on exosome concentration. (**F**) SERS intensity at 1580 cm^−1^ and NTA results depending on exosome concentration, with the statistical LOD indicated. In (**F**), one-way ANOVA with the correction of Tukey’s post hoc test for multiple comparisons. Bar graph data are presented as mean ± SD. ****P < 0.0001, ***P < 0.001, and **P < 0.01. Ab, antibody; ns, nonsignificant; n.d., nondetectable.

To evaluate the efficiency of antibody-free exosome capture, we confirmed exosome attachment to AZ-SERS substrates using scanning electron microscopy (SEM) and fluorescence imaging. SEM images revealed distinct gold-capped nanowire tip morphologies after gold deposition and effective exosome attachment (Fig. 2A and fig. S2). Fluorescence images showed markedly higher attachment to the fabricated substrate than to bare gold (Fig. 2B). This difference arises from the positively charged surface of ZnO, which promotes electrostatic interactions with negatively charged exosome membranes, whereas gold lacks such surface charge characteristics (*27*–*29*). This direct capture enabled stronger SERS signals by minimizing the distance between the exosomes and the plasmonic surface (*30*, *31*) (fig. S3, A and B). To ensure consistency with the experimental substrate, the finite-difference time-domain (FDTD) simulations were based on SEM-derived nanowire geometries and their statistical distributions (fig. S4). The simulations showed that the reported maximum enhancement factor (1.22 × 10^9^) corresponds to a localized hotspot confined to narrow nanowire gaps (fig. S5A). The effective near-field decays within several tens of nanometers from the nanowire surface. However, enhancement is spatially distributed around multiple neighboring nanowires in the lateral and vertical plane (figs. S5, B to D). Within this complex architecture, exosomes larger than individual hotspot regions can access multiple enhancement sites between tilted nanowires, enabling sufficient and reproducible SERS signal generation. Experimentally, the SERS signals under antibody-free conditions were fourfold stronger than those with antibody-functionalized substrates (Fig. 2C). Exosomes on our substrate exhibited a consistent peak at 1580 cm^−1^, corresponding to the amide II band, regardless of antibody modification (*32*, *33*). The signals were also highly uniform across the substrate, with a relative SD (RSD) of 4.17% (Fig. 2D and fig. S3C). Consistent with this interpretation, detergent-mediated membrane disruption using Triton X-100 markedly suppressed the exosome-associated SERS peak at 1580 cm^−1^, indicating that the detected signal predominantly originates from membrane-associated vesicular structures (fig. S6).

We next tested whether the US-SERS system could resolve differences in exosome concentrations, including determining the LOD. On the basis of these observations, the SERS spectra collected from serially diluted samples showed increased intensity with higher exosome levels (Fig. 2E). The signal intensity at 1580 cm^−1^ increased linearly with each 10-fold concentration step (*R*^2^ = 0.997) (Fig. 2F), enabling the indirect quantification of exosome abundance. Using the International Union of Pure and Applied Chemistry (IUPAC) definition under region of interest (ROI)–based measurement conditions, the statistical LOD for antibody-free detection was 69 particles/ml, while antibody-based detection exhibited a higher statistical LOD of 105 particles/ml with a comparable linear trend (fig. S3D). In contrast, NTA reliably measured only high concentrations (>10^7^ particles/ml) and became inaccurate or error-prone at lower ranges (Fig. 2F) (*8*).

However, the statistical LOD should not be interpreted as a physically validated particle-detection threshold because this value was extrapolated from the calibration curve and blank-signal variability (*34*–*36*). In the present ROI-based SERS measurement, the physical presence of exosomes within the fixed central ROI (25 × 25 μm^2^) is therefore a prerequisite for experimentally meaningful quantification at low concentrations. To evaluate this condition, we examined the spatial distribution of exosomes on AZ-SERS substrates (fig. S7, A to E). The hydrophobic AZ-SERS surface promoted wettability-dependent central accumulation, whereas plasma-induced hydrophilicity suppressed this effect, consistent with substrate-mediated trapping rather than diffusion-limited deposition (*37*, *38*). At 10^3^ particles/ml, exosome occupancy within the ROI was highly variable, consistent with Poisson-dominated fluctuations (*39*), while at 10^4^ particles/ml, reproducible enrichment within the central ROI was observed. SEM analysis under identical conditions confirmed the physical presence of exosomes.

We therefore separated the statistical LOD from experimentally supported low-concentration performance. Limit of blank (LoB)–positive detection was defined using a conservative 99% blank-threshold criterion, as $I_{\text{sample}}>I_{\text{blank}}+2.326\sigma_{\text{blank}}$, and the limit of quantitation (LOQ) was assigned based on reproducible signal precision (*40*). By this analysis, 10^3^ particles/ml was LoB-positive in 24 of 25 measurements but showed substantial variability (RSD > 50%), whereas 10^4^ particles/ml was LoB-positive in all 25 measurements with an RSD of 4.55% (fig. S7, F and G). Thus, 10^3^ particles/ml was defined as a LoB-positive detectable level, but not a quantitatively reliable level, whereas 10^4^ particles/ml was defined as the experimentally supported LOQ for AZ-SERS.

### Ultrasound-induced exosome enhancement and mechanistic insights

**Fig. 3.**
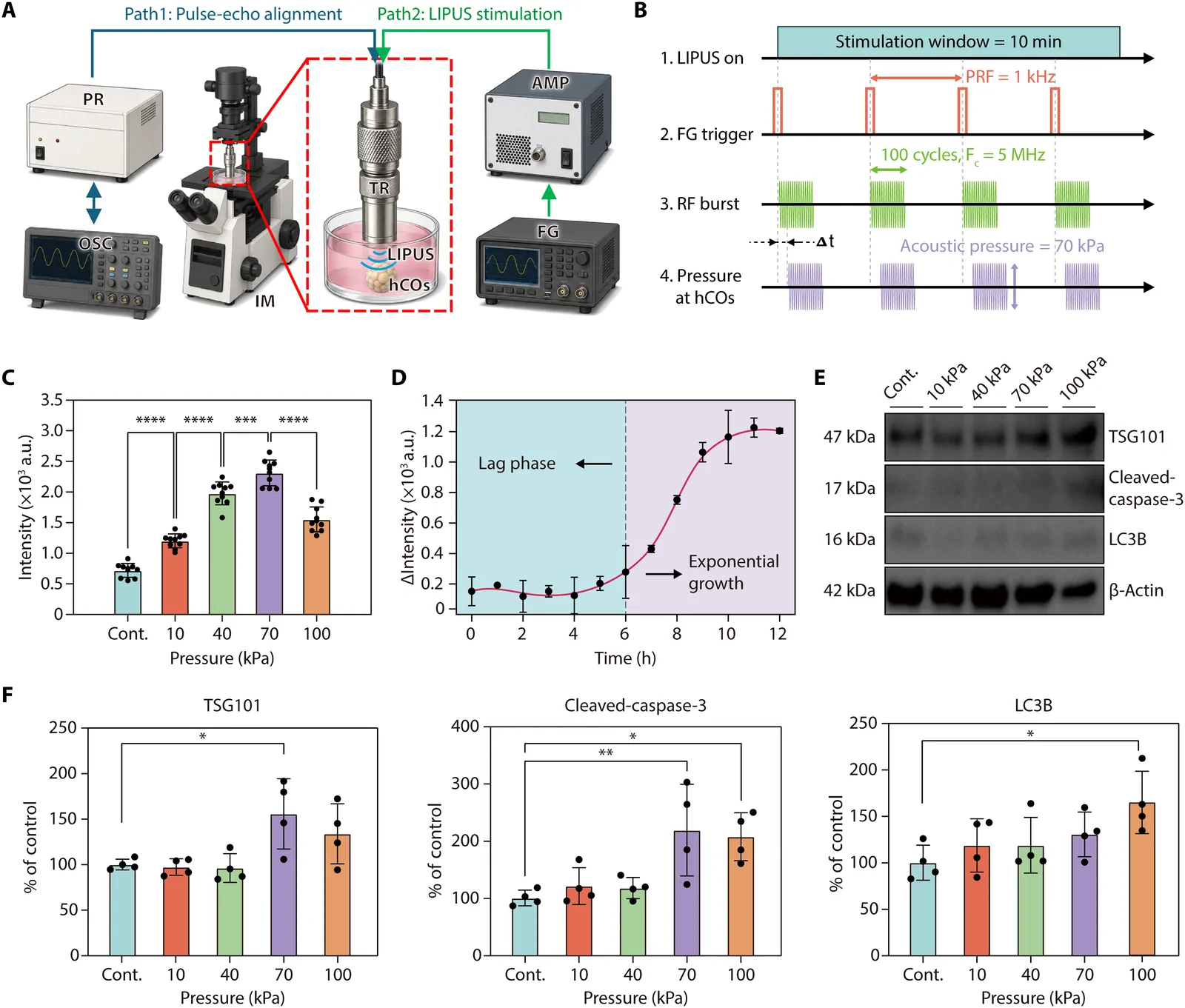
Optimization of ultrasound-induced exosome enhancement and its mechanistic insights. (**A**) Experimental setup of LIPUS stimulation applied to hCOs. (**B**) Schematic of the LIPUS stimulation sequence and acoustic parameters. (**C**) Exosome-associated SERS signal changes depending on acoustic pressure. One-way ANOVA with the correction of Tukey’s post hoc test for multiple comparisons. (**D**) Time course of exosome release following ultrasound exposure (*n* = 5). (**E**) Representative image of western blotting analysis of samples under different pressures. (**F**) Quantitative analysis of western blotting analysis of samples under different pressures. Markers for western blotting are TSG101, cleaved-caspase-3, and LC3B from left to right, respectively. One-way ANOVA with the correction of Dunnett’s post hoc test for multiple comparisons. Bar graph data are presented as mean ± SD. ****P < 0.0001, ***P < 0.001, **P < 0.01, and *P < 0.05. PR, pulser receiver; FG, function generator; AMP, amplifier; OSC, oscilloscope; IM, inverted microscope; TR, transducer; Cont., control.

We applied LIPUS stimulation under various acoustic parameters and sensitively detected changes in exosome secretion using AZ-SERS. An ultrasound transducer was immersed in the medium to directly deliver ultrasound to hCOs (Fig. 3A and fig. S8). With the stimulation time fixed at 10 min and the cycle number at 100 (Fig. 3B), we observed that exosome release increased with acoustic pressure, peaking at 70 kPa and decreasing at 100 kPa (Fig. 3C). When the acoustic pressure was fixed, varying the stimulation time and cycle number revealed that excessive acoustic energy suppressed exosome secretion (fig. S9, A and B). On the basis of these results, we designated 70 kPa, 10 min, and 100 cycles as the optimal parameters and performed all subsequent experiments under these conditions. Using these optimized parameters, an approximately 2.3-fold increase in exosome-associated SERS intensity was observed (fig. S9, C to E). NTA analysis confirmed that LIPUS stimulation did not alter the intrinsic properties of the exosomes (fig. S9, F and G). These results confirmed that LIPUS effectively enhanced exosome secretion without altering their physical characteristics.

We also monitored the dynamics of exosome release following ultrasound exposure (Fig. 3D and fig. S9H). A lag phase was observed for up to 6 hours, after which exosome secretion increased exponentially, suggesting the delayed activation of secretion-related pathways. To explore the underlying mechanisms, we examined the protein expression of ESCRT (TSG101), apoptosis (cleaved caspase-3), and autophagy (LC3B) (Fig. 3, E and F, and fig. S10). Stronger acoustic pressure was associated with increased ESCRT and apoptosis, which facilitate exosome biogenesis (*22*). However, higher-pressure conditions were also accompanied by increased autophagy, which may transiently limit exosome yield. This finding supports earlier observations that excessive ultrasound energy suppresses secretion (Fig. 3C and fig. S9, A and B). To further assess long-term adverse effects, quantitative apoptosis, neuronal marker, and viability analyses were performed at 24 and 48 hours poststimulation (fig. S11). These analyses showed a modest, transient increase in cleaved caspase-3 and terminal deoxynucleotidyl transferase–mediated (TUNEL) at 24 hours that normalized by 48 hours, with unchanged MAP2 expression and no loss of organoid viability.

### Aβ-associated signal analysis and APOE genotype classification

**Fig. 4.**
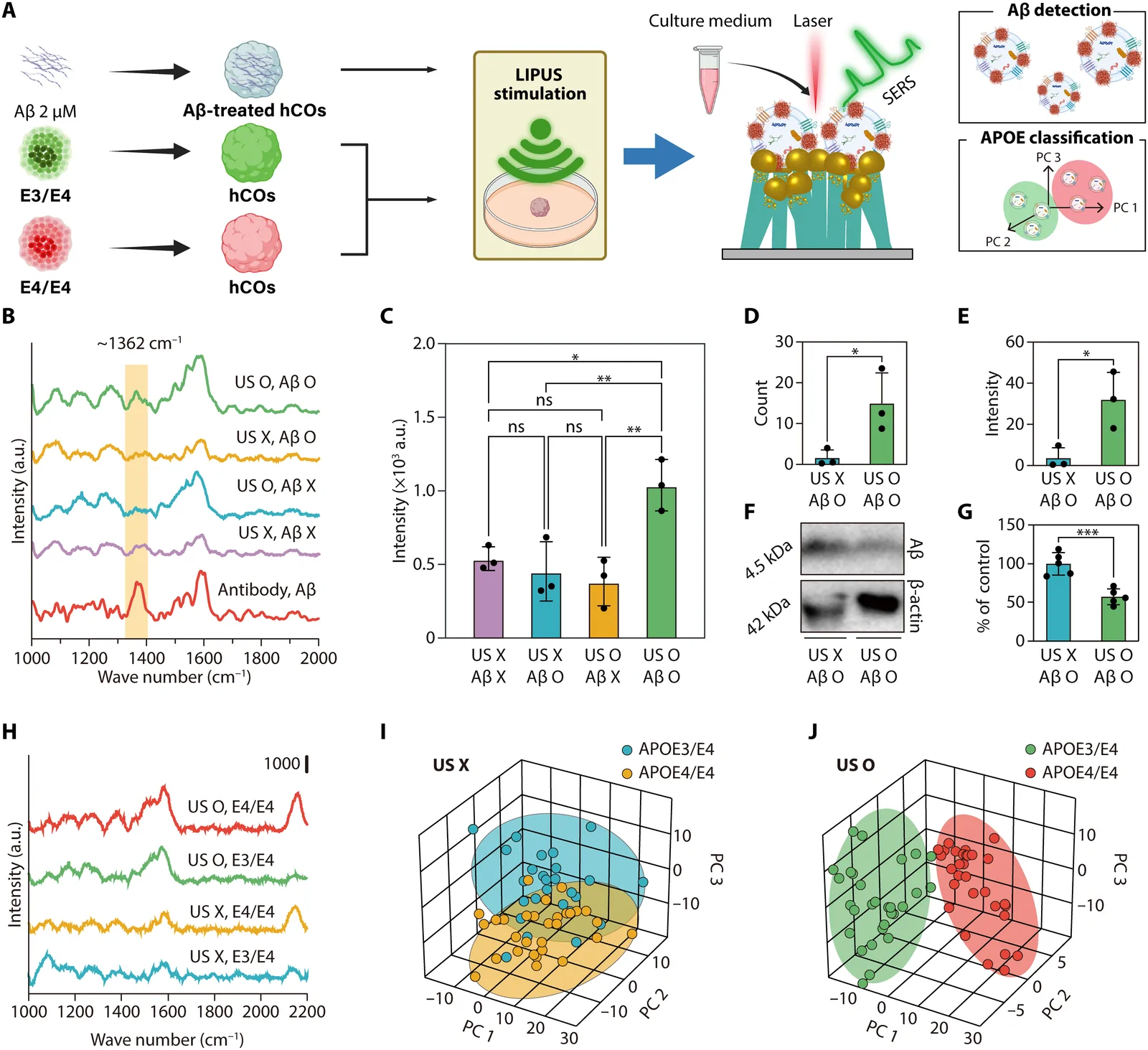
Biomarker detection and genotype classification using ultrasound-enhanced exosomes. (**A**) Schematic of validation experiment for biomarker detection and genotype classification. Aβ-treated hCOs and APOE-genotyped hCOs were stimulated with LIPUS, and subsequent signal analysis demonstrated Aβ detection and APOE classification. (**B**) SERS spectra of Aβ-treated and untreated hCOs with or without LIPUS stimulation (*n* = 3). (**C**) Quantification of the SERS signal at 1362 cm^−1^, which responds to Aβ treatment. One-way ANOVA with the correction of Tukey’s post hoc test for multiple comparisons. (**D**) Number of exosomes containing FITC-labeled Aβ. (**E**) Intensity of exosomes containing FITC-labeled Aβ. In (D) and (E), a two-tailed, unpaired *t* test was used. (**F**) Western blot showing Aβ and β-actin bands in hCOs with or without LIPUS stimulation. (**G**) Quantification of Aβ band intensity normalized to β-actin. A two-tailed, unpaired *t* test was used. (**H**) SERS spectra from exosomes of APOE E3/E4 and E4/E4 genotypes with and without ultrasound (*n* = 3). PCA of exosome signals according to APOE genotype without (**I**) and with (**J**) ultrasound. Bar graph data are presented as mean ± SD. ***P < 0.001, **P < 0.01, and *P < 0.05. Created in BioRender. Park, J. (2026) https://BioRender.com/4nvdvbu.

Using our US-SERS system, we validated that intact exosomal biomarkers could be sensitively detected and that multiplex analysis enabled the discrimination of genotype-dependent variations (Fig. 4A). We demonstrated its ability to detect changes associated with Aβ treatment and untreated hCOs carrying the E3/E4 genotype, and to classify APOE genotypes by distinguishing E3/E4 from E4/E4 hCOs.

Before evaluating Aβ-responsive exosomal signal, we first confirmed the spectral features of Aβ on the AZ-SERS substrate. Direct measurements of soluble Aβ on the developed substrate did not yield detectable signals at 1 nM, whereas Aβ was detected when captured via antibody functionalization (fig. S12A). These results indicate that the system preferentially detects exosome-associated Aβ rather than soluble Aβ, thereby minimizing matrix interference from nontarget, freely diffusing species. The SERS spectra of Aβ typically exhibit multiple characteristic peaks, among which our substrate with antibody primarily detected at 1362 cm^−1^, corresponding to C-H deformation (Fig. 4B and fig. S12, B and C) (*41*, *42*). Although antibody-mediated capture constrains the orientation of Aβ relative to the plasmonic surface, the 1362 cm^−1^ peak also increased when Aβ was deposited on the substrate from a dimethyl sulfoxide (DMSO) solution and was abolished upon washing, indicating that this peak covaries with the presence of Aβ (fig. S13). Consistent with this observation, analysis of the filtrate showed no detectable SERS signal above background (fig. S14), suggesting that residual free proteins, including soluble Aβ, do not substantially contribute to the observed spectra.

Next, we validated whether Aβ contained in exosomes could be detected on the antibody-free substrate. We compared Aβ-treated and untreated hCOs under both ultrasound-stimulated and unstimulated conditions (Fig. 4B). SERS analysis showed a negligible signal at 1362 cm^−1^ in Aβ-untreated hCOs, regardless of ultrasound treatment. Even in Aβ-treated hCOs, the signal remained weak without stimulation. However, when ultrasound was applied to Aβ-treated hCOs, a distinct peak at 1362 cm^−1^ was detected, with a roughly twofold increase in intensity compared to the unstimulated group (Fig. 4C). To investigate the basis of this signal increase, we labeled Aβ with fluorescein isothiocyanate (FITC) and analyzed exosome-bound fluorescence via NTA (Fig. 4, D and E, and fig. S15). In the absence of ultrasound, even Aβ-treated hCOs secreted fewer than five detectable exosomes. In contrast, under LIPUS stimulation, the number of fluorescence-detected exosomes increased markedly to approximately 15. Total fluorescence intensity of FITC-labeled exosomes was about nine times higher in the ultrasound group, indicating that enhanced signal of Aβ resulted from increased exosome secretion. Accordingly, the increase in exosome release suggests that more Aβ was exported from the hCOs, and we confirmed a concomitant reduction in intracellular Aβ levels (Fig. 4, F and G, and fig. S16). These results suggested that the US-SERS system is well suited for the detection of biomarker proteins located on the surface of intact exosomes.

Leveraging this capability, we explored whether exosomal profiles could be used to distinguish APOE genotypes. In AD, individuals with the APOE E4/E4 genotype exhibit greater amyloid accumulation and neuroinflammation than those with the E3/E4 genotype, indicating a higher pathological risk (*6*, *43*). We collected exosomes from the hCOs of each genotype and compared the SERS signals with and without LIPUS stimulation. Although slight differences in the spectral peaks were observed, no single peak provided sufficient specificity for reliable interpretation (Fig. 4H). Instead, PCA enabled effective differentiation by capturing subtle distributed variations across the spectra. Nonetheless, as our system captured composite signals from multiple biomarkers simultaneously, we applied PCA to the SERS spectrum (Fig. 4, I and J). PCA is a multivariate statistical analysis tool that highlights dissimilarities and patterns, thereby enabling the extraction of valuable information from complex SERS spectra (*44*). The elbow plot indicated that the optimal number of principal components was three, as determined by second-derivative analysis (fig. S17) (*45*). Therefore, we conducted three-dimensional PCA by calculating the silhouette score, an indicator of PCA performance (*46*). Without ultrasound, silhouette scores were 0.37, a value within the range of 0.25 to 0.5, indicating weak separation. However, the ultrasound-enhanced exosomes yielded a silhouette score of 0.5, reflecting clear and distinguishable genotype-specific clustering. Together, these findings demonstrated that ultrasound enhancement improved the sensitivity of intact exosome SERS profiling and enabled the distinction of subtle pathological variations according to genotype using multivariate spectral analysis.

### Ultrasound-induced transcriptomic changes in exosome-associated genes and pathways

**Fig. 5.**
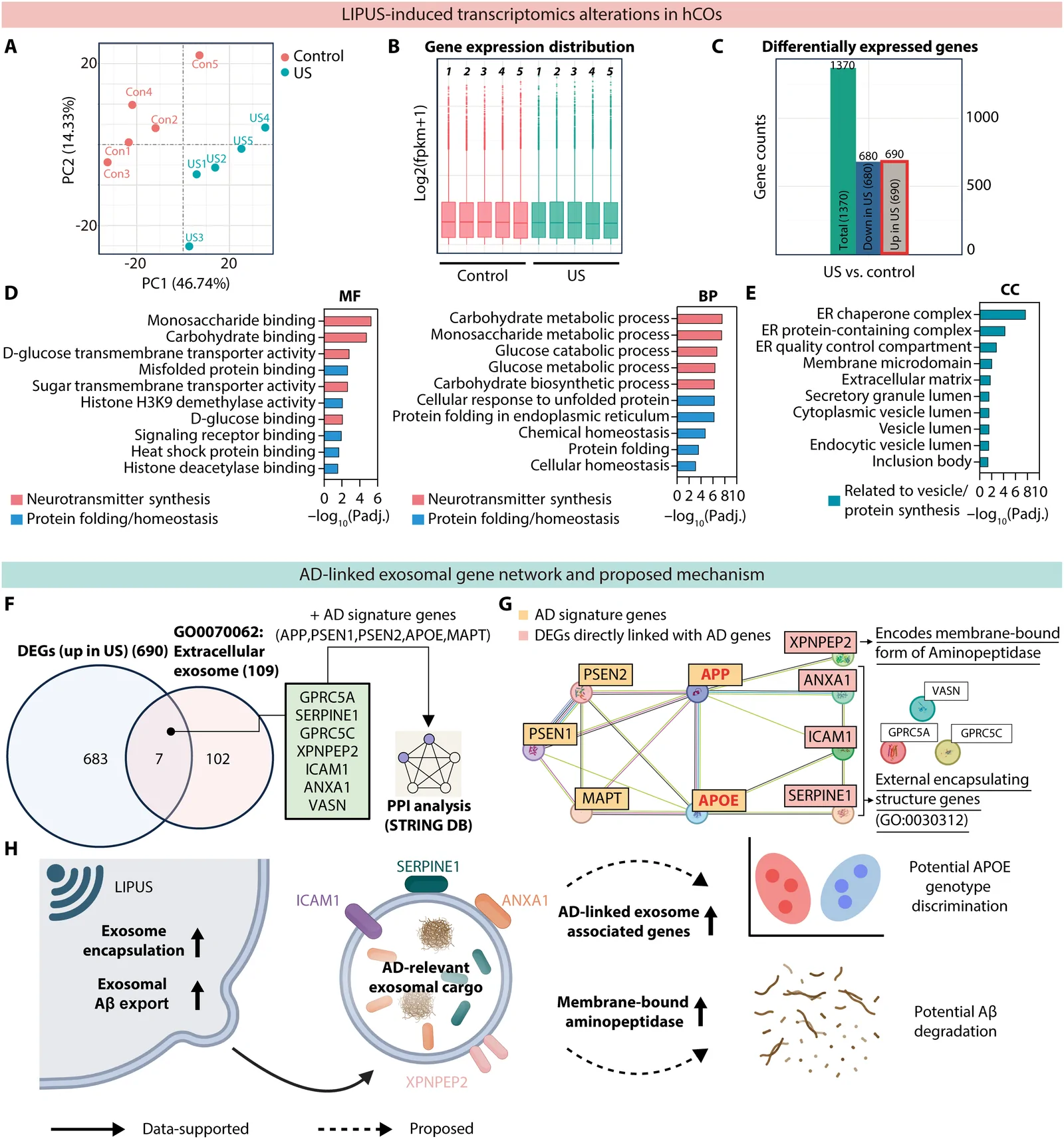
RNA sequencing analysis comparing transcriptomic profiles of organoids with and without LIPUS stimulation. (**A**) PCA to evaluate intragroup differences. (**B**) Gene expression distribution box plot. *X* axis represents the name of the samples, and *Y* axis indicates the log_2_(FPKM+1). Parameters of box plots are indicated including upper quartile, mid-value, low quartile, maximum, and minimum. (**C**) Number of DEGs including up-regulation and down-regulation. Total *n* = 1370, up-regulated *n* = 690, down-regulated *n* = 680. (**D**) Gene ontology analysis (molecular function, MF; biological process, BP). *P* values were adjusted using the Benjamini-Hochberg correction for controlling the false discovery rate (FDR). (**E**) GO analysis (cellular component, CC). *P* values were adjusted using the Benjamini-Hochberg correction for controlling the FDR. (**F**) Coexpression Venn diagram between up-regulated DEGs (*n* = 690) and extracellular exosome-related genes (*n* = 109; GO0070062). Seven genes overlapped and were used for PPI analysis (STRING database) with AD signature genes. (**G**) PPI analysis results. Four genes (ICAM1, SERPINE1, ANXA1, and XPNPEP2) were directly linked with AD signature genes (APP and APOE). (**H**) Hypothetical summary of this study. Created in BioRender. Park, J. (2026) https://BioRender.com/4rmeebr.

Following LIPUS stimulation, we observed an increase in exosome production accompanied by an enhanced release of biomarkers (Figs. 3 and 4). To validate these findings and identify the underlying genes and pathways, we performed transcriptomic profiling using RNA sequencing. PCA demonstrated a clear separation between the control and LIPUS-stimulated groups, indicating substantial transcriptomic divergence (Fig. 5A). Quality control of the sequencing data was confirmed through gene expression distribution, FPKM distribution, and Pearson correlation analysis (Fig. 5B and fig. S18). Differential expression analysis identified 1370 differentially expressed genes (DEGs), with 690 up-regulated and 680 down-regulated (Fig. 5C).

Next, gene ontology (GO) enrichment analysis was performed using up-regulated DEGs. We found alterations in pathways related to neurotransmitter synthesis and protein folding in both the molecular function (MF) and biological process (BP) categories, highlighting changes in processes such as monosaccharide binding, carbohydrate metabolism, and cellular responses to unfolded proteins (Fig. 5D). Cellular component (CC) analysis further indicated that these DEGs were primarily associated with extracellular components and protein synthesis (Fig. 5E). Collectively, these findings suggest that LIPUS stimulation enhances protein and neurotransmitter synthesis and promotes secretion events linked to exosomal pathways.

To investigate this phenomenon in detail, we intersected the 690 up-regulated DEGs with the GO category “extracellular exosome” (GO:0070062). Seven overlapping genes were identified: GPRC5A, SERPINE1, GPRC5C, XPNPEP2, ICAM1, ANXA1, and VASN (Fig. 5F). To determine whether these genes were related to AD, we performed a protein-protein interaction (PPI) analysis incorporating five AD signature genes (APP, PSEN1, PSEN2, APOE, and MAPT) using the STRING database (Fig. 5G). This network revealed that four of the identified genes (ICAM1, SERPINE1, ANXA1, and XPNPEP2) were functionally connected to AD signature genes, particularly APOE and APP (*47*, *48*). Three of these genes (ICAM1, SERPINE1, and ANXA1) belong to the GO category “external encapsulating structure” (GO:0030312), whereas XPNPEP2 encodes the membrane-bound form of aminopeptidase P. Aminopeptidase is capable of hydrolyzing and degrading Aβ peptides (*49*). Together, these data support a model in which LIPUS stimulation promotes exosome release via pathways associated with APOE and APP. The reduction in Aβ observed in our earlier experiments (Fig. 4, B to G) may thus result from proteolytic degradation of Aβ at the exosomal surface or from exosomal secretion of Aβ into the extracellular space. A hypothetical summary of this mechanism is presented in Fig. 5H.

To assess whether the ultrasound-induced transcriptomic changes are reflected in the SERS measurements, we examined which spectral features dominantly contributed to the PCA separation and compared them with reference SERS spectra of AD-associated exosomal proteins encoded by the up-regulated genes (fig. S19). Several of these PCA-dominant bands overlapped with prominent Raman features observed in purified ICAM1, SERPINE1, and ANXA1 spectra measured on the same substrate. In particular, shared bands around 1234 and 1370 cm^−1^ were consistently detected across all three proteins and coincided with regions of high PCA loading. Although direct one-to-one assignment of individual Raman peaks to specific proteins is not feasible in label-free intact exosome SERS, this correspondence supports the interpretation that transcriptomically up-regulated, exosome-associated AD-related proteins contribute to the PCA-driving composite SERS fingerprints beyond effects attributable to exosome concentration alone.

## DISCUSSION

In this study, we present a US-SERS system that combines ultrasound-induced exosome enhancement with antibody-free SERS detection. By noninvasively stimulating exosome secretion from hCOs and directly capturing intact vesicles on an AZ-SERS substrate, our approach overcomes the limitation of low vesicle abundance and enables sensitive, label-free multiplex SERS profiling. Using hCOs made it possible to perform RNA sequencing–based mechanistic analysis, which is challenging in heterogeneous patient samples, and revealed the ultrasound-induced activation of exosome genes linked to APOE and APP. This strategy allowed us to resolve subtle APOE genotype–dependent variations in exosomal signals, underscoring its potential as a clinically relevant noninvasive diagnostic tool for AD. This clinical relevance is further supported by a simple serum-based assessment showing that exosome-derived SERS fingerprints remain dominant in the composite SERS response even in a complex biological matrix (fig. S20). However, cellular responses to LIPUS are highly context-dependent, indicating that acoustic parameters require cell-specific optimization rather than universal application (fig. S21).

To evaluate the performance of our system, we quantified key enhancement metrics at multiple stages of the detection pipeline. First, the engineered AZ-SERS substrate facilitated antibody-free exosome capture, achieving an extrapolated statistical LOD estimate of 69 particles/ml compared with 105 particles/ml for antibody-functionalized substrates, and an experimentally supported LOQ of 10^4^ particles/ml, below the practical NTA measurement range (>10^7^ particles/ml) (Fig. 2 and fig. S7). Second, LIPUS stimulation resulted in an approximately 2.3-fold increase in exosome-associated SERS intensity in hCO-derived samples, supporting enhanced detectability under optimized conditions (Fig. 3 and fig. S9). Third, in Aβ-treated models, ultrasound further enhanced the intensity of a SERS feature at 1362 cm^−1^ that reproducibly increased in response to Aβ treatment and ultrasound-induced exosome secretion, with an approximately twofold enhancement compared to unstimulated conditions (Fig. 4, B and C). Last, by applying PCA to the SERS spectra of exosomes from different APOE genotypes, we achieved accurate classification with improved silhouette scores from 0.37 (baseline) to 0.5 (ultrasound-enhanced), demonstrating the system’s potential for genotype-level stratification (Fig. 4, H to J). These metrics underscored the robust enhancement capability of our approach in terms of both physical vesicle production and signal-level detectability.

Beyond diagnostics, our findings suggest a potential therapeutic implication of ultrasound in facilitating Aβ clearance through exosomal pathways. Upon LIPUS stimulation, hCOs exhibited a marked reduction in Aβ, accompanied by increased release of Aβ-carrying exosomes into the extracellular environment (Fig. 4, B to G). This effect was validated by both fluorescence-labeled NTA measurements and Western blotting of Aβ levels. These observations indicated that ultrasound not only enhanced biomarker accessibility, but may also modulate pathological protein dynamics by promoting exosomal export. Consistent with our transcriptomic analysis (Fig. 5, D to G), the reduction in Aβ may result from both proteolytic degradation of Aβ at the exosomal surface and increased exosomal secretion into the extracellular space, as summarized in Fig. 5H. Together, our results suggest that noninvasive ultrasound protocols could be leveraged to reduce amyloid accumulation via enhanced exosomal secretion, offering a complementary strategy for AD treatment.

At the same time, several points warrant cautious interpretation. The extrapolated LOD should be regarded as a statistical sensitivity estimate, whereas 10^4^ particles/ml represents the experimentally supported LOQ under the ROI-based SERS measurement conditions. In addition, the optimized LIPUS condition did not cause overt cellular damage (fig. S11), and control experiments support that the SERS signals were not dominated by soluble biomolecules or free protein contaminants (figs. S6 and S14). Nevertheless, because acoustic stimulation can elicit context-dependent stress responses, minor contributions from stress-associated or apoptotic vesicles should be interpreted with caution. The transcriptomic data provide pathway-level mechanistic insight, but do not directly demonstrate protein-level changes in exosomal cargo. Moreover, we interpret the enhanced antibody-free SERS response as a distributed multisite plasmonic effect within the three-dimensional AZ-SERS architecture (fig. S5), and caution is needed when attributing the signal to amplification from a single narrow nanogap. Thus, these considerations define the interpretive boundaries of LIPUS-enhanced, vesicle-associated SERS profiling.

## MATERIALS AND METHODS

### Cell culture

Human iPSCs were subcultured as described by Park *et al.* (*50*). iPSCs (E3/E4, BIONi010-C; E4/E4, BIONi010-C-4; European Bank for Induced Pluripotent Stem Cells) were cultured on 100-mm plates coated with Matrigel (Corning Matrigel hESC-Qualified Matrix, Corning, USA) in Dulbecco’s minimum essential medium (DMEM)/F-12 with GlutaMAX (10565018, Gibco, USA) for 1 hour at room temperature. After coating, the solution was aspirated and 4 ml of mTeSR medium supplemented with Y-27632 (Abcam, UK) was added. One day after seeding, the medium was replaced with mTeSR Plus (mTeSR^+^; ST100-0276, StemCell Technologies, Canada) without Y and was refreshed daily. When confluence reached >70%, the cells were passaged using ReLeSR (ST05872, StemCell Technologies, Canada). The detached colonies were collected in the mTeSR^+^ medium and centrifuged at 300*g* for 2 min at room temperature. The pellet was resuspended in 3 ml of mTeSR^+^, and 300 μl of the suspension was seeded into a new Matrigel-coated plate and incubated at 37°C with 5% CO_2_.

Before culturing the U87 cells on the 35-mm confocal dish (no. 100350, SPL, Korea), the cell adhesion area (confocal area) was coated with 300 μl of poly-d-lysine (100 μg/ml; P0089, Merck, Germany) in deionized (DI) water. The confocal dish was incubated at 37°C with 5% CO_2_ for 30 min, then directly aspirated and washed five to six times with Dulbecco’s phosphate-buffered saline (DPBS), respectively. Cells (1 × 10^5^) were seeded onto the confocal area and incubated with the culture medium composed of high-glucose DMEM (SH3024.01, Cytiva, Korea), 10% qualified fetal bovine serum (#26140079, Thermo Fisher Scientific, USA), and penicillin/streptomycin (100 U/ml and 100 μg/ml; P4333, Merck, Germany). LIPUS was applied when the cell confluency reached over 70% and the culture medium was collected 20 hours after LIPUS stimulation for exosome purification.

### iPSCs-derived hCO generation

The generation of hCOs followed the same protocol as that for the iPSC subculture process (*50*). hCOs were generated from human iPSCs using AggreWell 800 plates (34811, StemCell Technologies, Canada). Single iPSCs (1.5 × 10^6^) were seeded, centrifuged, and cultured in EB medium supplemented with Y-27632 (ab144494, Abcam, UK) at 37°C, 5% CO_2_ (day 0). On day 1, medium was replaced with EB medium without Y. From day 2, cells were cultured in induction medium containing dorsomorphin (10 μM; P5499, Merck, Germany), SB431542 (10 μM; 1614, TOCRIS, UK), and 2-mercaptoethanol (0.1 mM; 21985023, Gibco, USA) in DMEM/F-12 with GlutaMAX (10565018, Gibco, USA), KnockOut Serum Replacement (20%; A3181501, Gibco, USA), MEM NonEssential Amino Acids (1%; 11140050, Gibco, USA), and penicillin/streptomycin (100 U/ml and 100 μg/ml; P4333, Merck, Germany). The induction medium was replaced once on day 4 with the same medium.

On day 6, the cultures were switched to Neurobasal-A medium (10888-022, Gibco, USA) supplemented with GlutaMAX (35050061, Gibco, USA), B-27 Supplement without vitamin A (12587010, Gibco, USA), and penicillin/streptomycin and further enriched with Matrigel Basement Membrane (0.5% v/v; 354234, Corning, USA), epidermal growth factor (EGF; 01-017, Merck, Germany), and fibroblast growth factor (FGF; 233-FB, R&D Systems, USA). Aggregates were collected and transferred to 100-mm petri dishes with 14 ml of NB+ medium containing basement membrane Matrigel, EGF, and FGF, and incubated on an orbital shaker at 90 rpm overnight.

On day 7, EBs were transferred into 96-well ultralow attachment U-bottom plates (7007, Corning, USA) with 200 μl of NB+ medium containing Matrigel, EGF, and FGF per well. The medium was changed daily until day 15, and every other day until day 24. From days 25 to 42, the NB+ medium was supplemented with BDNF (20 ng/ml; 450-02, Peprotech, USA) and NT-3 (20 ng/ml; 450-03, Peprotech, USA), with media changed every other day. On day 43, each hCO was transferred to 24-well ultralow attachment plates (3473, Corning, USA) with 500 μl of NB+ medium per well, refreshed every 4 days. Mature hCOs (>day 80) were used in the experiments.

### Aβ treatment for AD-associated condition

Aβ (4014447, Bachem, Switzerland) and FITC-conjugated Aβ (4033502, Bachem, Switzerland) were fully dissolved in 1 ml of 1,1,1,3,3,3-hexafluoro-2-propanol (HFIP; 105228, Merck, Germany) to ensure the formation of complete monomers and incubated in the dark on a rocker at room temperature for 72 hours, as previously described (*47*). After incubation, the Aβ solution in HFIP was aliquoted into 50 μl portions into individual EP tubes. The aliquoted Aβ in HFIP was lyophilized using the SpeedVac SPD1030 Integrated Vacuum Concentrator. The lyophilized Aβ was stored at −80°C and later reconstituted to a concentration of 100 μM using anhydrous DMSO (276855, Merck, Germany) and sterile DPBS to prepare stock solutions. hCOs were treated at a final concentration of 2 μM in a 37°C, 5% CO_2_ incubator for 20 hours, and were used after removing the medium containing Aβ. LIPUS stimulation was then applied, and 20 hours after the stimulation was completed, both the media and hCOs were harvested for analysis.

### Exosome preparation and NTA

After LIPUS stimulation was applied to the hCOs, 1 ml of culture media and the hCOs themselves were collected from each sample. The collected culture media were centrifuged at 3000*g* for 10 min at 4°C to remove cell debris, and the supernatant was used for exosome isolation. Exosomes were isolated using size exclusion chromatography with an IZON Gen 2 qEV 35-nm original series column. From the isolated EVs, 1 ml was separated for NTA. NTA was conducted using an NS300 (Malvern) instrument equipped with a 642-nm laser, without a filter to measure the concentration, intensity, and size distribution of exosomes. In experiments involving FITC-conjugated Aβ, measurements using the 642-nm laser module were also performed without a filter, whereas a 500-nm blue-range filter was applied for measurements using the 488-nm laser module. Each sample was measured independently using both laser modules.

### SERS substrate fabrication

The gold-coated ZnO-nanowire SERS substrate was fabricated as previously described (*14*). In brief, to prepare the ZnO seed layer, 25 mM zinc acetate dihydrate [Zn(CH_3_COO)_2_ · 2H_2_O, 96459, Sigma-Aldrich] in isopropanol with sonication to prevent aggregation was spin coated on a cleaned silicon wafer with 3000 rpm for 30 s and preheated at 100°C on a hot plate for 1 min, which was repeated 10 times. After the ZnO seed layer was baked at 350°C for 1 hour to remove any remaining organic solvents, ZnO nanowires were synthesized using the hydrothermal synthesis method. The ZnO seeded substrate was immersed in a solution containing 40 mM zinc nitrate hexahydrate [Zn(NO_3_)_2_ · 6H_2_O, 228737, Sigma Aldrich] and 25 mM hexamethylenetetramine (33233, Sigma-Aldrich), with the reaction surface facing downward to avoid ZnO crystal accumulation on the seed layer. Hydrothermal synthesis was then performed at 90°C for 1 hour to create ZnO nanowires. After the ZnO nanowire substrate was washed with distilled water and dried with nitrogen gas, a 100-nm gold layer was deposited on the substrate using a thermal evaporator.

### FDTD simulation

Electromagnetic simulations of our SERS substrate were performed using FDTD simulations to evaluate the enhancement factors. A 3D computer-aided design (CAD)–driven FDTD simulation, previously introduced in the literature (*14*, *51*), was adopted to realistically represent a randomly oriented SERS substrate. The modeling process involved defining the structural parameters of individual nanowires using CAD and subsequently assigning material properties to mimic the AZ-SERS substrate. The optical constants for ZnO and gold were obtained from the literature (*52*, *53*). To ensure accurate field resolution near the nanostructure, a mesh override of 1 nm was applied within a 0.4 μm × 0.4 μm × 0.65 μm region embedded in the overall 0.4 μm × 0.4 μm × 2.1 μm simulation region. A 785-nm total-field scattered-field light source was used for the excitation of the nanostructures.

### SERS measurement

To compare the SERS signal intensity between the antibody-functionalized and antibody-free conditions, the SERS substrates were first functionalized with an anti-CD81 antibody. Half-antibody fragments were functionalized to immobilize the antibodies onto the gold-coated SERS substrate via gold-thiol binding. First, the antibodies were diluted to 0.05 mg/ml in PBS (P3813, Sigma-Aldrich) and mixed with 0.02 mM tris(2-carboxyethyl)phosphine (TCEP; Bond-Breaker TCEP Solution, 77720, Thermo Fisher Scientific) at a 1:1 volume ratio. The mixture was incubated at room temperature for 1 hour to allow efficient cleavage into half-antibody fragments via disulfide bond reduction (*54*). After the reaction, 10 μl of TCEP-treated antibody solution was dropped on the SERS substrate and incubated at 4°C for overnight. After the functionalized SERS substrate was washed with DI water for 15 min twice and dried with nitrogen gas, 10 μl of exosome solution was dropped onto the SERS substrate. DI water was used for washing to minimize salt-induced interference during SERS measurements, under conditions that preserved vesicle integrity (fig. S22) (*55*).

For quantifying exosomes under antibody-free conditions, 10 μl of exosome solution extracted from human hCOs were incubated on the SERS substrates at 37°C for 2 hours. After incubation, the substrate was washed twice with DI water for 15 min and dried with nitrogen gas. In addition, to evaluate the correlation between the SERS signal of Aβ-containing exosomes and Aβ, the SERS spectra of Aβ were also measured. For measuring the SERS signal of Aβ, the half anti-Aβ antibody fragments were used, which were generated and immobilized on the substrates following the same procedure used for half anti-CD81 antibody fragments functionalization. A total of 10 μl of each concentration of Aβ solution was incubated on the functionalized substrate at 4°C for 1 hour, followed by washing with DI water for 15 min twice and drying with nitrogen gas. To examine the potential contribution of transcriptomically up-regulated exosome-associated proteins to the SERS signals, reference SERS spectra of ICAM1, SERPINE1, and ANXA1 were acquired on the same AZ-SERS substrate under antibody-free conditions (table S1).

The SERS spectra were collected by Raman spectroscopy (NS 200, Nanoscope Systems) with 10 mW of a 785-nm laser through a 50× objective lens (numerical aperture, 0.8). The laser spot (diameter ∼10 μm; effective area ∼78.5 μm^2^) was fully contained within the predefined central ROI. For each substrate, 25 spectra were acquired from a 5 × 5 grid within this ROI, with an acquisition time of 1 s per single shot. The signals were preprocessed for baseline correction using an asymmetric least-squares method and smoothed with a Savitzky-Golay filter. For quantitative analysis, the 10 spectra with the highest mutual cross-correlation among the 25 grid spectra were averaged to generate one representative ROI-level spectrum, reducing hotspot-dependent spatial variability. The extrapolated statistical LOD was calculated according to the IUPAC definition as $y_{\text{LOD}}=\left(y_{\text{blank}}+3.3\sigma_{\text{blank}}\right)$, where $y_{\text{blank}}$ and $\sigma_{\text{blank}}$ represent the mean and SD of the substrate-only blank signal, respectively (*34*). The corresponding concentration was then estimated from the log-linear calibration curve as ${\text{log}}_{10}C_{\text{LOD}}=\left(y_{\text{LOD}}-\text{intercept}\right)/\text{slope}$.

### LIPUS stimulation

The acoustic properties of an ultrasound transducer (V326-SU, Evident) were characterized using pulse-echo and hydrophone measurements (fig. S23) as previously described (*56*, *57*). The transducer was positioned over the hCOs by adjusting the *X* and *Y* axes using a three-axis linear motor stage (L-836, PI). The focal depth along the *Z* axis was aligned using a pulser/receiver (DPR 500, JSR). Ultrasound signals were generated using a function generator (33600A, Keysight), amplified using an RF amplifier (325LA, Electronics & Innovation), and delivered to transducer. A center frequency of 5 MHz and pulse repetition frequency of 1 kHz were used for stimulation.

### Western blotting

Western blotting was performed as previously described (*58*). The hCOs were stored on ice throughout the procedure. Following LIPUS stimulation, the samples were washed three times with DPBS and lysed in RIPA buffer supplemented with a protease and phosphatase inhibitor cocktail (PPPi; P8340, Merck, Germany). Cells were homogenized, sonicated, and centrifuged at 13,000 rpm at 4°C to collect the supernatants. Protein concentration was measured using a BCA assay. A standard curve was generated using bovine serum albumin (A7906, Merck, Germany) at concentrations of 0, 1, 2, and 4 mg/ml. Standards and samples were incubated at 37°C with 200 μl of BCA working solution containing 4% CuSO_4_ for 15 min before absorbance measurement. Equal amounts of protein (6 μg in 18 μl of mixture composed with RIPA + PPPi, sample buffer, protein stock) were denatured at 95°C for 5 min and loaded on Bolt bis-tris gels (NW001505, Thermo Fisher Scientific, USA) with Novex Sharp Pre-stained Protein Standard (LC5800, Thermo Fisher Scientific, USA). Electrophoresis was performed at 200 V for 22 min, followed by transfer to polyvinylidene difluoride membranes using a semidry system (100 V, 1 hour). The membranes were blocked with 5% skim milk in tris-buffered saline with Tween 20 (TBST), incubated with primary antibodies overnight at 4°C, washed with TBST, and probed with horseradish peroxidase–conjugated secondary antibodies. Signals were detected using ECL substrate and imaged using the iBright system (Thermo Fisher Scientific, USA).

### Cryosection and immunohistochemistry

Cryosectioning and immunohistochemistry were performed as previously described (*50*). After LIPUS stimulation, hCOs were washed with PBS, fixed in 4% paraformaldehyde in PBS for 24 hours, dehydrated in 30% sucrose for 72 hours, embedded in OCT compound (Sakura, USA), and frozen at −80°C. Samples were sectioned at 25 μm thickness using a cryotome (Leica, Germany) and mounted on slides. For immunohistochemistry, sections were permeabilized with 0.3% Triton X-100 (Triton X-100; X100-5mL, Merck, Germany) in DPBS (PBST) and blocked with 5% normal horse serum (Vector Laboratories, USA) in 0.3% PBST. Primary antibodies (table S2) were applied overnight at 4°C. The next day, the slides were washed and incubated with fluorescence-conjugated secondary antibodies for 1 hour at room temperature in the dark. After washing, the sections were mounted with Gel/Mount Mounting Solution (MO1, Biomeda, USA) and imaged using a Thunder Imaging System (Leica, Germany).

### TUNEL assay

To assess apoptosis following LIPUS stimulation in hCO, a TUNEL assay was performed using presectioned hCO samples. The Click-iT TUNEL Alexa Fluor Imaging Assay for Microscopy & HCS (C10247, Thermo Fisher Scientific, USA) was used according to the manufacturer’s instructions. Cryosectioned hCO slides were washed with DI water and permeabilized with 0.3% Triton X-100 in DPBS. Samples were then blocked with 3% bovine serum albumin (BSA) in DPBS. The TdT reaction and Click-iT reaction were performed following the manufacturer’s protocol. After the TUNEL labeling, samples were washed twice with 3% BSA in DPBS for 2 min each. Primary and secondary antibody labeling was subsequently performed using the same immunostaining conditions.

### MTT assay

Cell viability of hCOs following LIPUS stimulation was assessed using an MTT assay. At 24 and 48 hours after stimulation, organoids were incubated with MTT solution. A MTT stock solution (5 mg/ml) was added to reach a final concentration of 0.5 mg/ml in culture medium. Organoids were incubated with the MTT-containing medium at 37°C in a humidified atmosphere with 5% CO_2_ for 6 hours. After incubation, samples were carefully washed twice with DPBS and completely lysed in DMSO. The lysates were further incubated at 37°C for 2 hours to ensure complete solubilization of formazan crystals. Absorbance was measured at 570 nm using a spectrophotometer.

### RNA sequencing analysis

Total RNA was isolated from organoids with and without LIPUS stimulation. For RNA extraction, two hCOs were pooled to generate one biological sample. Following the isolation, library preparation and sequencing were performed using Novogene (Beijing, China). The mRNA was enriched using poly-T oligo–attached magnetic beads, fragmented, and converted into cDNA. Strand-specific libraries were generated using end repair, adaptor ligation, polymerase chain reaction amplification, and purification. Libraries were quantified and sequenced on Illumina platforms based on sequencing by synthesis to produce paired-end reads. Clean reads were aligned to the human reference genome (hg38) using HISAT2, and gene expression levels were quantified using feature counts. DEGs were identified using DESeq2 (|log_2_FC| ≥ 1, adjusted *P* ≤ 0.05). GO analysis was performed using ToppGene and PPI networks were analyzed using the STRING database.

### Human serum experiment

Human serum samples were provided by Korea Dementia Research Center (KDRC, Distribution ID: 25KDRC-A001), and their use was approved by the Institutional Review Board of Sungkyunkwan University (approval number: 2024-70-070). Written informed consent was obtained from the participant in accordance with institutional guidelines. The samples were collected from a cognitively normal subject (APOE3/E4) with amyloid plaque deposition in the brain. Both serum samples and serum-derived purified exosomes were processed and measured under the same AZ-SERS conditions as described above.

### Statistical analysis

All statistical analyses and data visualizations were performed using GraphPad Prism, and some schematic illustrations were created using BioRender.com. Data are presented as mean ± SD. All data points were included in the analysis, with statistical details provided in the corresponding figure legends, except for the MTT assay (fig. S11C), in which one control value was excluded based on Grubbs’ test for outlier detection (α = 0.05). Experiments involving platform characterization and analytical optimization, such as AZ-SERS development, acoustic parameter optimization, and exosome secretion monitoring, were performed using technical replicates, whereas all other experiments were conducted with biological replicates.

## References

[1] V. S. LeBleu, R. Kalluri, Exosomes as a multicomponent biomarker platform in cancer. Trends Cancer 6, 767–774 (2020). 10.1016/j.trecan.2020.03.007 32307267

[2] I. S. Nila, D. M. Sumsuzzman, Z. A. Khan, J. H. Jung, A. S. Kazema, S. J. Kim, Y. Hong, Identification of exosomal biomarkers and its optimal isolation and detection method for the diagnosis of Parkinson’s disease: A systematic review and meta-analysis. Ageing Res. Rev. 82, 101764 (2022). 10.1016/j.arr.2022.101764 36273807

[3] W. Yu, J. Hurley, D. Roberts, S. K. Chakrabortty, D. Enderle, M. Noerholm, X. O. Breakefield, J. K. Skog, Exosome-based liquid biopsies in cancer: Opportunities and challenges. Ann. Oncol. 32, 466–477 (2021). 10.1016/j.annonc.2021.01.074 PMC8268076 33548389

[4] A. Khalyfa, D. Gozal, Exosomal miRNAs as potential biomarkers of cardiovascular risk in children. J. Transl. Med. 12, 162 (2014). 10.1186/1479-5876-12-162 PMC4057926 24912806

[5] K. Yuyama, H. Sun, S. Mitsutake, Y. Igarashi, Sphingolipid-modulated exosome secretion promotes clearance of amyloid-beta by microglia. J. Biol. Chem. 287, 10977–10989 (2012). 10.1074/jbc.M111.324616 PMC3322859 22303002

[6] C.-C. Liu, C.-C. Liu, T. Kanekiyo, H. Xu, G. Bu, Apolipoprotein E and Alzheimer disease: Risk, mechanisms and therapy. Nat. Rev. Neurol. 9, 106–118 (2013). 10.1038/nrneurol.2012.263 PMC3726719 23296339

[7] W. Oosthuyzen, N. E. Sime, J. R. Ivy, E. J. Turtle, J. M. Street, J. Pound, L. E. Bath, D. J. Webb, C. D. Gregory, M. A. Bailey, J. W. Dear, Quantification of human urinary exosomes by nanoparticle tracking analysis. J. Physiol. 591, 5833–5842 (2013). 10.1113/jphysiol.2013.264069 PMC3872755 24060994

[8] G. Kowkabany, Y. Bao, Nanoparticle tracking analysis: An effective tool to characterize extracellular vesicles. Molecules 29, 4672 (2024). 10.3390/molecules29194672 PMC11477862 39407601

[9] E. Dezhakam, B. Khalilzadeh, M. Mahdipour, I. Isildak, H. Yousefi, M. Ahmadi, A. Naseri, R. Rahbarghazi, Electrochemical biosensors in exosome analysis; a short journey to the present and future trends in early-stage evaluation of cancers. Biosens. Bioelectron. 222, 114980 (2023). 10.1016/j.bios.2022.114980 36521207

[10] C. Z. J. Lim, Y. Zhang, Y. Chen, H. Zhao, M. C. Stephenson, N. R. Y. Ho, Y. Chen, J. Chung, A. Reilhac, T. P. Loh, C. L. H. Chen, H. Shao, Subtyping of circulating exosome-bound amyloid beta reflects brain plaque deposition. Nat. Commun. 10, 1144 (2019). 10.1038/s41467-019-09030-2 PMC6408581 30850633

[11] S. Song, J. U. Lee, M. J. Jeon, S. Kim, S. J. Sim, Detection of multiplex exosomal miRNAs for clinically accurate diagnosis of Alzheimer’s disease using label-free plasmonic biosensor based on DNA-Assembled advanced plasmonic architecture. Biosens. Bioelectron. 199, 113864 (2022). 10.1016/j.bios.2021.113864 34890883

[12] Z. Wang, S. Zong, Y. Wang, N. Li, L. Li, J. Lu, Z. Wang, B. Chen, Y. Cui, Screening and multiple detection of cancer exosomes using an SERS-based method. Nanoscale 10, 9053–9062 (2018). 10.1039/c7nr09162a 29718044

[13] Y. Liu, M. Li, H. Liu, C. Kang, C. Wang, Cancer diagnosis using label-free SERS-based exosome analysis. Theranostics 14, 1966–1981 (2024). 10.7150/thno.92621 PMC10945334 38505618

[14] Y. Jo, Y. Kim, R. Kang, S. Lee, D. D. Nguyen, S. Park, D. Lee, J. W. Han, I. Mook-Jung, L. P. Lee, J. C. Park, I. Kim, Monitoring the dynamics of Alzheimer’s disease biomarkers and the APOE-tau axis via human cerebral organoids with immuno-SERS. Adv. Sci. 12, e05660 (2025). 10.1002/advs.202505660 PMC12463057 40444455

[15] K. Liang, F. Liu, J. Fan, D. Sun, C. Liu, C. J. Lyon, D. W. Bernard, Y. Li, K. Yokoi, M. H. Katz, E. J. Koay, Z. Zhao, Y. Hu, Nanoplasmonic quantification of tumor-derived extracellular vesicles in plasma microsamples for diagnosis and treatment monitoring. Nat. Biomed. Eng. 1, 0021 (2017). 10.1038/s41551-016-0021 PMC5543996 28791195

[16] S. Dong, Y. Wang, Z. Liu, W. Zhang, K. Yi, X. Zhang, X. Zhang, C. Jiang, S. Yang, F. Wang, X. Xiao, Beehive-inspired macroporous SERS probe for cancer detection through capturing and analyzing exosomes in plasma. ACS Appl. Mater. Interfaces 12, 5136–5146 (2020). 10.1021/acsami.9b21333 31894690

[17] H. Shin, B. H. Choi, O. Shim, J. Kim, Y. Park, S. K. Cho, H. K. Kim, Y. Choi, Single test-based diagnosis of multiple cancer types using exosome-SERS-AI for early stage cancers. Nat. Commun. 14, 1644 (2023). 10.1038/s41467-023-37403-1 PMC10039041 36964142

[18] N. P. Hessvik, A. Llorente, Current knowledge on exosome biogenesis and release. Cell. Mol. Life Sci. 75, 193–208 (2018). 10.1007/s00018-017-2595-9 PMC5756260 28733901

[19] A. Bobrie, M. Colombo, G. Raposo, C. Thery, Exosome secretion: Molecular mechanisms and roles in immune responses. Traffic 12, 1659–1668 (2011). 10.1111/j.1600-0854.2011.01225.x 21645191

[20] A. Savina, M. Furlán, M. Vidal, M. I. Colombo, Exosome release is regulated by a calcium-dependent mechanism in K562 cells. J. Biol. Chem. 278, 20083–20090 (2003). 10.1074/jbc.M301642200 12639953

[21] Y. He, S. Yang, P. Liu, K. Li, K. Jin, R. Becker, J. Zhang, C. Lin, J. Xia, Z. Ma, Z. Ma, R. Zhong, L. P. Lee, T. J. Huang, Acoustofluidic interfaces for the mechanobiological secretome of MSCs. Nat. Commun. 14, 7639 (2023). 10.1038/s41467-023-43239-6 PMC10665559 37993431

[22] L. A. Ambattu, S. Ramesan, C. Dekiwadia, E. Hanssen, H. Li, L. Y. Yeo, High frequency acoustic cell stimulation promotes exosome generation regulated by a calcium-dependent mechanism. Commun. Biol. 3, 553 (2020). 10.1038/s42003-020-01277-6 PMC7536404 33020585

[23] Q. Zeng, S. Hong, X. Wang, Y. Cheng, J. Sun, W. Xia, Regulation of exosomes secretion by low-intensity pulsed ultrasound in lung cancer cells. Exp. Cell Res. 383, 111448 (2019). 10.1016/j.yexcr.2019.05.029 31152706

[24] J. Yoo, D. Heo, Y. Hwang, C. Kim, B. Park, Ultrasound-mediated membrane modulation for biomedical applications. Nanomaterials 15, 884 (2025). 10.3390/nano15120884 PMC12196113 40559247

[25] Y.-C. Yoon, K.-S. Park, S.-D. Kim, Effects of low preheating temperature for ZnO seed layer deposited by sol–gel spin coating on the structural properties of hydrothermal ZnO nanorods. Thin Solid Films 597, 125–130 (2015).

[26] S. Boubenia, A. S. Dahiya, G. Poulin-Vittrant, F. Morini, K. Nadaud, D. Alquier, A facile hydrothermal approach for the density tunable growth of ZnO nanowires and their electrical characterizations. Sci. Rep. 7, 15187 (2017). 10.1038/s41598-017-15447-w PMC5680254 29123216

[27] T. Yasui, T. Yanagida, S. Ito, Y. Konakade, D. Takeshita, T. Naganawa, K. Nagashima, T. Shimada, N. Kaji, Y. Nakamura, I. A. Thiodorus, Y. He, S. Rahong, M. Kanai, H. Yukawa, T. Ochiya, T. Kawai, Y. Baba, Unveiling massive numbers of cancer-related urinary-microRNA candidates via nanowires. Sci. Adv. 3, e1701133 (2017). 10.1126/sciadv.1701133 PMC5744465 29291244

[28] T. Yasui, P. Paisrisarn, T. Yanagida, Y. Konakade, Y. Nakamura, K. Nagashima, M. Musa, I. A. Thiodorus, H. Takahashi, T. Naganawa, T. Shimada, N. Kaji, T. Ochiya, T. Kawai, Y. Baba, Molecular profiling of extracellular vesicles via charge-based capture using oxide nanowire microfluidics. Biosens. Bioelectron. 194, 113589 (2021). 10.1016/j.bios.2021.113589 34543824

[29] K. Chattrairat, T. Yasui, S. Suzuki, A. Natsume, K. Nagashima, M. Iida, M. Zhang, T. Shimada, A. Kato, K. Aoki, F. Ohka, S. Yamazaki, T. Yanagida, Y. Baba, All-in-one nanowire assay system for capture and analysis of extracellular vesicles from an ex vivo brain tumor model. ACS Nano 17, 2235–2244 (2023). 10.1021/acsnano.2c08526 PMC9933609 36655866

[30] M. Moskovits, Surface-enhanced spectroscopy. Rev. Mod. Phys. 57, 783–826 (1985).

[31] H. Shin, S. Oh, D. Kang, Y. Choi, Protein quantification and imaging by surface-enhanced Raman spectroscopy and similarity analysis. Adv. Sci. 7, 1903638 (2020). 10.1002/advs.201903638 PMC7284192 32537409

[32] H. Shin, H. Jeong, J. Park, S. Hong, Y. Choi, Correlation between cancerous exosomes and protein markers based on surface-enhanced Raman spectroscopy (SERS) and principal component analysis (PCA). ACS Sens. 3, 2637–2643 (2018). 10.1021/acssensors.8b01047 30381940

[33] H. J. Koster, T. Rojalin, A. Powell, D. Pham, R. R. Mizenko, A. C. Birkeland, R. P. Carney, Surface enhanced Raman scattering of extracellular vesicles for cancer diagnostics despite isolation dependent lipoprotein contamination. Nanoscale 13, 14760–14776 (2021). 10.1039/d1nr03334d PMC8447870 34473170

[34] J. Mocak, A. M. Bond, S. Mitchell, G. Scollary, A statistical overview of standard (IUPAC and ACS) and new procedures for determining the limits of detection and quantification: Application to voltammetric and stripping techniques (technical report). Pure Appl. Chem. 69, 297–328 (1997).

[35] D. M. Rissin, C. W. Kan, T. G. Campbell, S. C. Howes, D. R. Fournier, L. Song, T. Piech, P. P. Patel, L. Chang, A. J. Rivnak, E. P. Ferrell, J. D. Randall, G. K. Provuncher, D. R. Walt, D. C. Duffy, Single-molecule enzyme-linked immunosorbent assay detects serum proteins at subfemtomolar concentrations. Nat. Biotechnol. 28, 595–599 (2010). 10.1038/nbt.1641 PMC2919230 20495550

[36] X. Zhang, M. A. Young, O. Lyandres, R. P. Van Duyne, Rapid detection of an anthrax biomarker by surface-enhanced Raman spectroscopy. J. Am. Chem. Soc. 127, 4484–4489 (2005). 10.1021/ja043623b 15783231

[37] M. Sperling, M. Gradzielski, Droplets, evaporation and a superhydrophobic surface: Simple tools for guiding colloidal particles into complex materials. Gels 3, 15 (2017). 10.3390/gels3020015 PMC6318646 30920512

[38] H. Gelderblom, C. Diddens, A. Marin, Evaporation-driven liquid flow in sessile droplets. Soft Matter 18, 8535–8553 (2022). 10.1039/d2sm00931e PMC9682619 36342336

[39] M. Lee, D. Kim, T. Y. Heo, T. Park, W. Kim, D. Choi, H. Kim, J. Kim, Universal inherent fluctuations in statistical counting of large particles in slurry used for semiconductor manufacturing. Sci. Rep. 10, 14731 (2020). 10.1038/s41598-020-71768-3 PMC7477581 32895450

[40] L. A. Currie, Limits for qualitative detection and quantitative determination. Application to radiochemistry. Anal. Chem. 40, 586–593 (1968).

[41] A. V. Krasnoslobodtsev, T. Deckert-Gaudig, Y. Zhang, V. Deckert, Y. L. Lyubchenko, Polymorphism of amyloid fibrils formed by a peptide from the yeast prion protein Sup35: AFM and Tip-enhanced Raman scattering studies. Ultramicroscopy 165, 26–33 (2016). 10.1016/j.ultramic.2016.03.011 PMC4879610 27060278

[42] G. V. Pavan Kumar, B. A. Ashok Reddy, M. Arif, T. K. Kundu, C. Narayana, Surface-enhanced Raman scattering studies of human transcriptional coactivator p300. J. Phys. Chem. B 110, 16787–16792 (2006). 10.1021/jp063071e 16913819

[43] T. Hashimoto, A. Serrano-Pozo, Y. Hori, K. W. Adams, S. Takeda, A. O. Banerji, A. Mitani, D. Joyner, D. H. Thyssen, B. J. Bacskai, M. P. Frosch, T. L. Spires-Jones, M. B. Finn, D. M. Holtzman, B. T. Hyman, Apolipoprotein E, especially apolipoprotein E4, increases the oligomerization of amyloid beta peptide. J. Neurosci. 32, 15181–15192 (2012). 10.1523/JNEUROSCI.1542-12.2012 PMC3493562 23100439

[44] M. Greenacre, P. J. F. Groenen, T. Hastie, A. I. D’Enza, A. Markos, E. Tuzhilina, Principal component analysis. Nat. Rev. Methods Primers 2, 100 (2022).

[45] R. Perry, S. Panigrahi, J. Bien, D. Witten, Inference on the proportion of variance explained in principal component analysis. arXiv:2402.16725 [stat.ME] (2024).

[46] P. J. Rousseeuw, Silhouettes: A graphical aid to the interpretation and validation of cluster analysis. J. Comput. Appl. Math. 20, 53–65 (1987).

[47] J. C. Park, S. H. Baik, S. H. Han, H. J. Cho, H. Choi, H. J. Kim, H. Choi, W. Lee, D. K. Kim, I. Mook-Jung, Annexin A1 restores Aβ_1-42_-induced blood–brain barrier disruption through the inhibition of RhoA-ROCK signaling pathway. Aging Cell 16, 149–161 (2017). 10.1111/acel.12530 PMC5242298 27633771

[48] A. G. Bryant, M. Hu, B. C. Carlyle, S. E. Arnold, M. P. Frosch, S. Das, B. T. Hyman, R. E. Bennett, Cerebrovascular senescence is associated with tau pathology in Alzheimer’s disease. Front. Neurol. 11, 575953 (2020). 10.3389/fneur.2020.575953 PMC7525127 33041998

[49] M. J. Dhanavade, K. D. Sonawane, Insights into the molecular interactions between aminopeptidase and amyloid beta peptide using molecular modeling techniques. Amino Acids 46, 1853–1866 (2014). 10.1007/s00726-014-1740-0 24729013

[50] J. C. Park, S. Y. Jang, D. Lee, J. Lee, U. Kang, H. Chang, H. J. Kim, S. H. Han, J. Seo, M. Choi, D. Y. Lee, M. S. Byun, D. Yi, K. H. Cho, I. Mook-Jung, A logical network-based drug-screening platform for Alzheimer’s disease representing pathological features of human brain organoids. Nat. Commun. 12, 280 (2021). 10.1038/s41467-020-20440-5 PMC7804132 33436582

[51] Y. Jo, Y. Kim, S. Lee, K. Chung, I. Kim, Plasmonic field enhancement effect of tip sharpness on bowtie structure with curved side arm. Micro Nano Syst. Lett. 13, 18 (2025).

[52] M. R. Querry, Optical constants, Contractor Report CRDC-CR-85034 (1985).

[53] P. B. Johnson, R. W. Christy, Optical constants of the noble metals. Phys. Rev. B 6, 4370–4379 (1972).

[54] H. Sharma, R. Mutharasan, Half antibody fragments improve biosensor sensitivity without loss of selectivity. Anal. Chem. 85, 2472–2477 (2013). 10.1021/ac3035426 23356211

[55] J. Liu, S. Srivastava, T. Li, F. Moujane, J. Y. Lee, Y. Chen, H. Liu, S. X. Deng, Y. H. Xie, On the feasibility of SERS-based monitoring of drug loading efficiency in exosomes for targeted delivery. Biosensors 15, 141 (2025). 10.3390/bios15030141 PMC11939968 40136938

[56] J. Yoo, H. Kim, Y. Kim, H. G. Lim, H. H. Kim, Collapse pressure measurement of single hollow glass microsphere using single-beam acoustic tweezer. Ultrason. Sonochem. 82, 105844 (2022). 10.1016/j.ultsonch.2021.105844 PMC8799605 34965507

[57] J. Yoo, J. Kim, J. Lee, H. H. Kim, Red blood cell trapping using single-beam acoustic tweezers in the Rayleigh regime. iScience 26, 108178 (2023). 10.1016/j.isci.2023.108178 PMC10616376 37915606

[58] J. C. Park, J. W. Han, W. Lee, J. Kim, S. E. Lee, D. Lee, H. Choi, J. Han, Y. J. Kang, Y. N. Diep, H. Cho, R. Kang, W. J. Yu, J. Lee, M. Choi, S. W. Im, J. I. Kim, I. Mook-Jung, Microglia gravitate toward amyloid plaques surrounded by externalized phosphatidylserine via TREM2. Adv. Sci. 11, e2400064 (2024). 10.1002/advs.202400064 PMC11425970 38981007

