# Supplementary material for: Ultrasound-enhanced exosome secretion and antibody-free SERS profiling for Alzheimer’s disease via iPSC-derived cortical organoids
Source: Sci Adv. 2026 Sep 23;12(39):eaed3930. doi: 10.1126/sciadv.aed3930 (PMC13600212; doi:10.1126/sciadv.aed3930)
Supplement: Supplementary file 1 [file sciadv.aed3930_sm.pdf]

Science Advances
AAAS

Supplementary Materials for

Ultrasound-enhanced exosome secretion and antibody-free SERS profiling for Alzheimer's disease via iPSC-derived cortical organoids

Jinhee Yoo *et al.*

Corresponding author: Luke P. Lee, lplee@bwh.harvard.edu; Jong-Chan Park, jongchan@g.skku.edu; Inki Kim, inki.kim@skku.edu; Byullee Park, byullee@skku.edu

Sci. Adv. **12**, eaed3930 (2026)
DOI: 10.1126/sciadv.aed3930

This PDF file includes:

Figs. S1 to S23
Tables S1 and S2

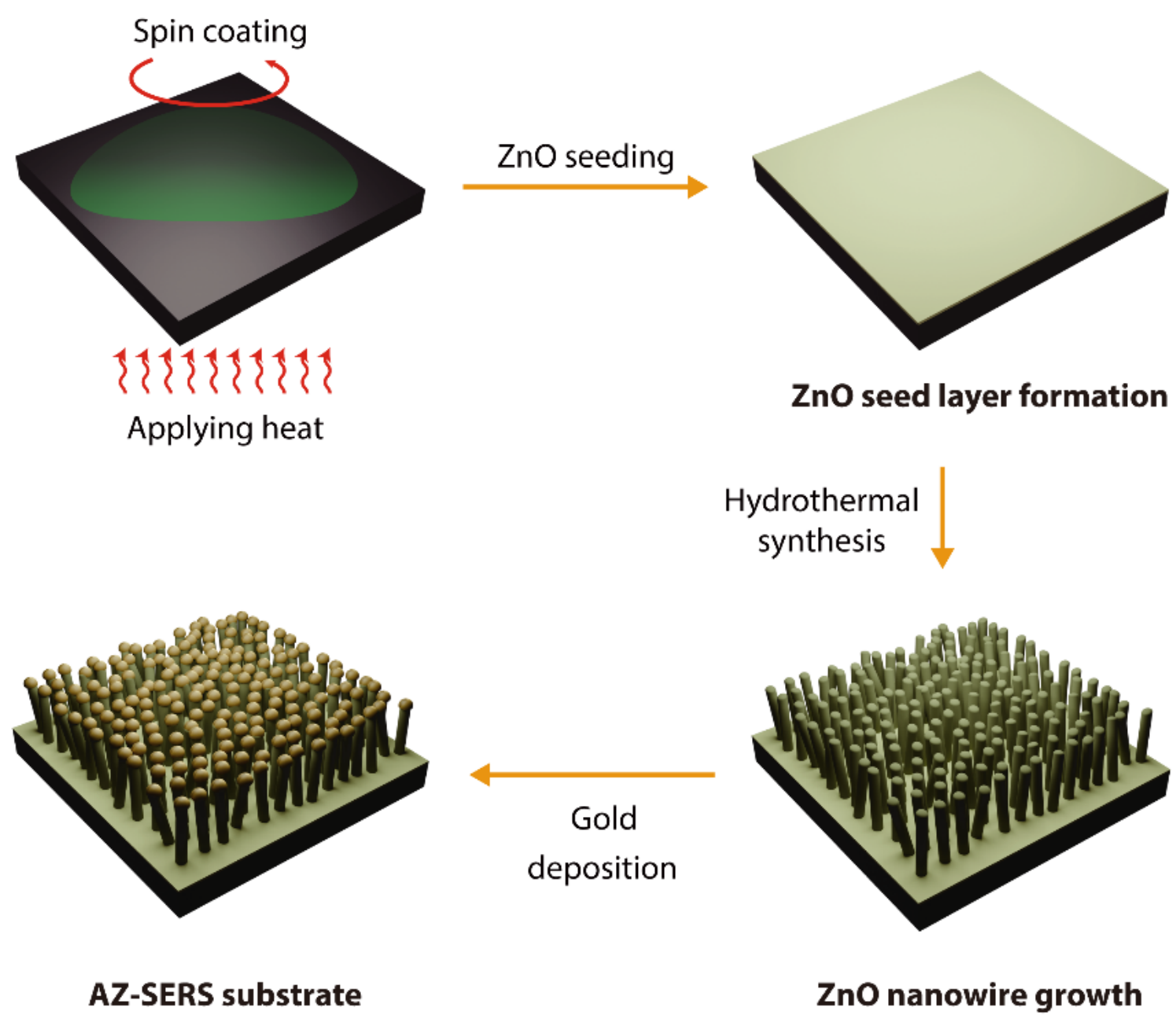

Fig. S1. Schematic of AZ-SERS substrate fabrication. ZnO seed layers were formed by repeated spin coating and heating, followed by hydrothermal ZnO nanowire growth and gold deposition.

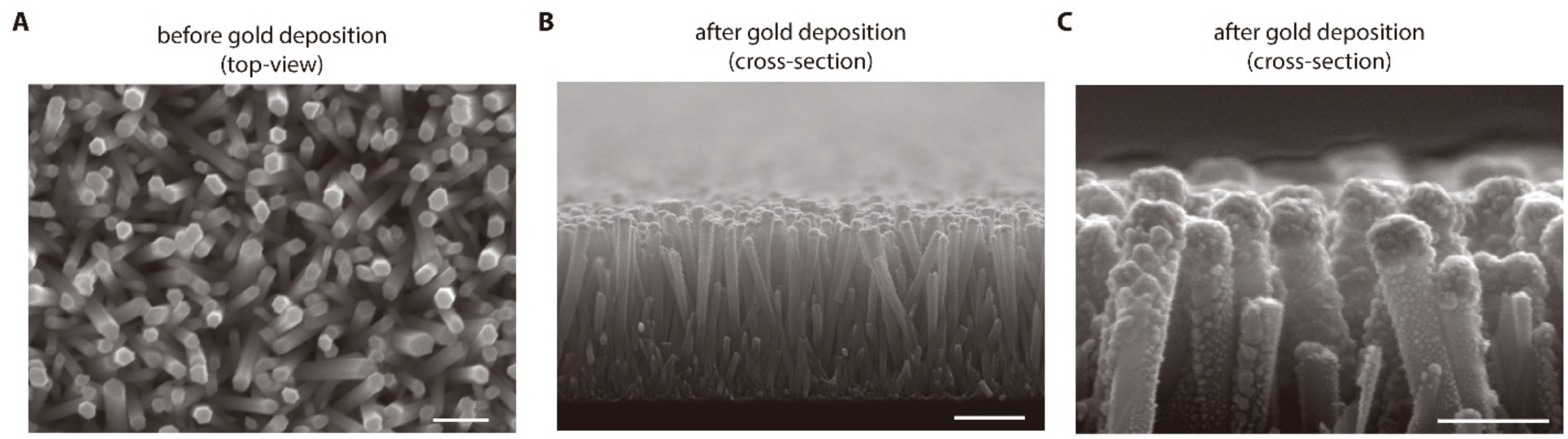

Fig. S2. SEM characterization of the AZ-SERS substrate. (A) Top-view SEM image of the AZ-SERS substrate showing densely distributed ZnO nanowires before gold deposition. Scale bar, 200 nm. **(B)** Low-magnification cross-sectional SEM image of the AZ-SERS substrate showing densely packed ZnO nanowires. Scale bar, 500 nm. **(C)** High-magnification cross-sectional SEM image highlighting the nanowire tips and sidewalls, clearly confirming the presence of a continuous gold coating deposited. Scale bar, 200 nm.

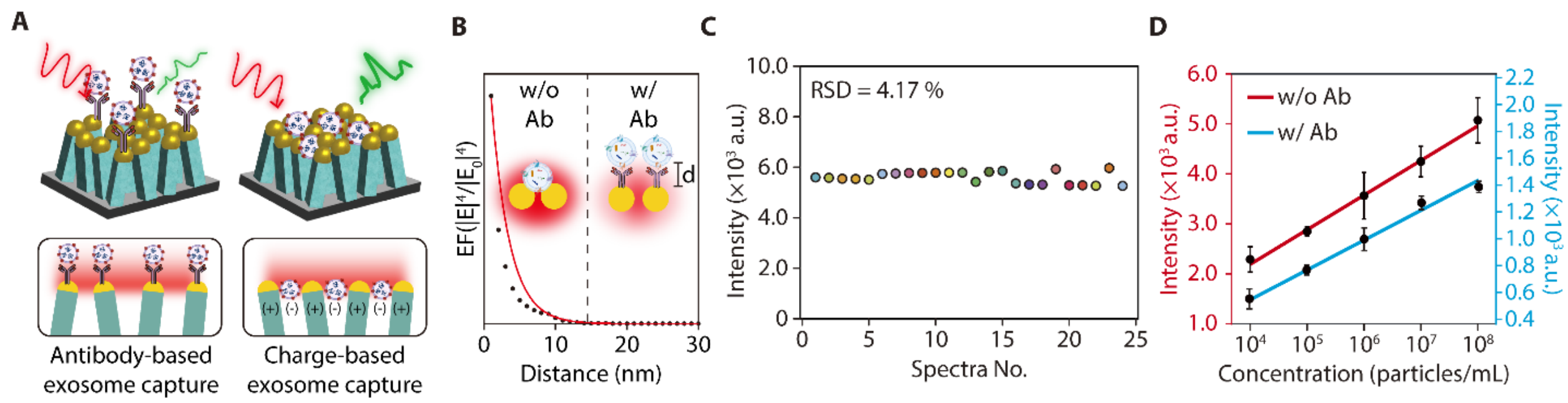

Fig. S3. Capture mechanism, uniformity, and linearity of the AZ-SERS substrate. (A) Schematic comparison of antibody-based and charge-based exosome capture on the AZ-SERS substrate. **(B)** Comparison of near-field coupling with and without antibody-mediated spacing on the AZ-SERS substrate. **(C)** Uniformity of exosomal SERS signals across different measurement points on the AZ-SERS substrate. (**D)** Linear correlation between exosome concentration and SERS intensity for antibody-free and antibody-functionalized substrates. The antibody-functionalized substrate shows a statistical LOD of 105 particles/mL. Data are presented as mean ± s.d.

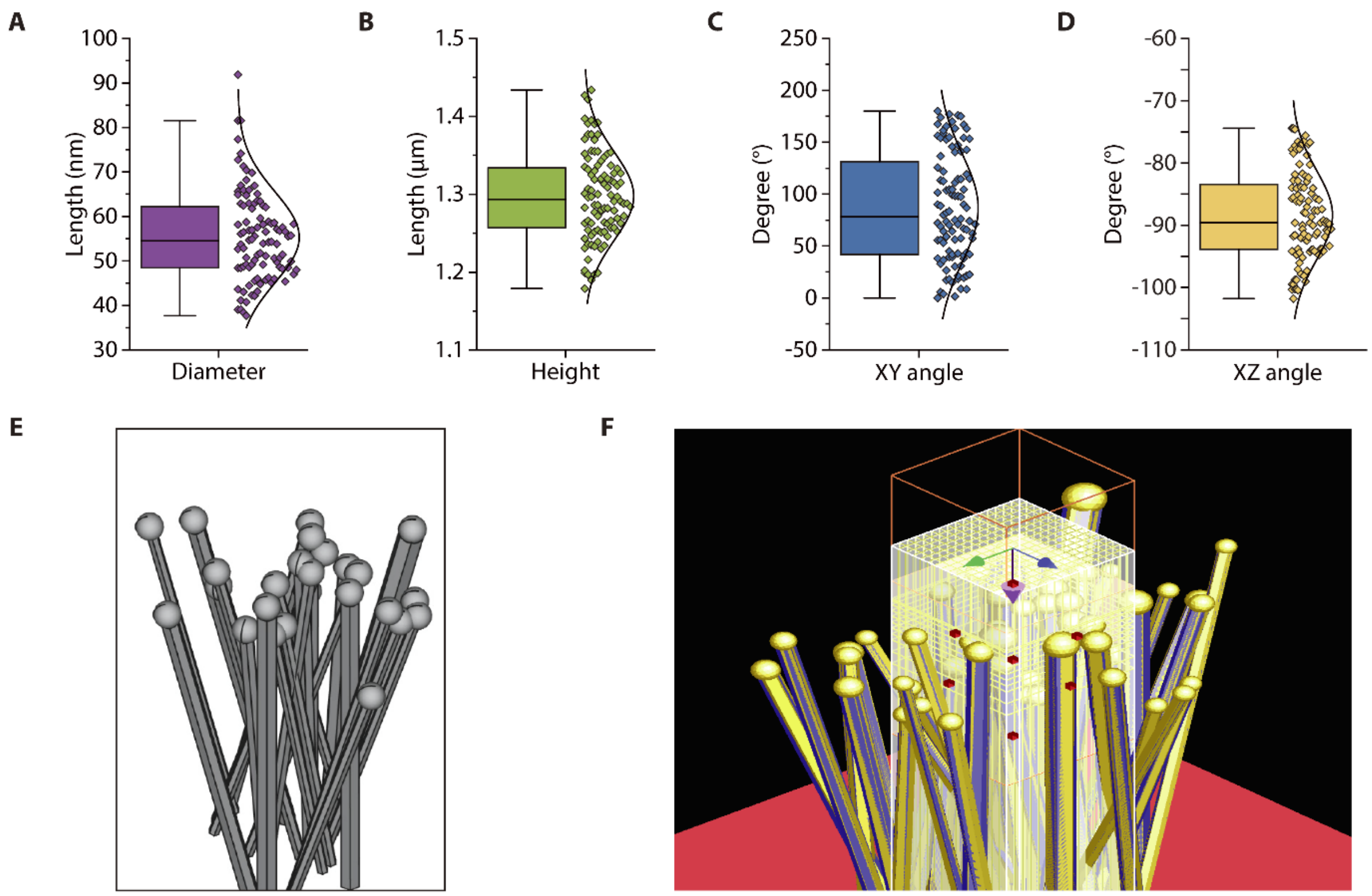

Fig. S4. SEM-derived parameter extraction and FDTD simulation geometry. (A–D) Statistical distributions of nanowire structural parameters extracted from SEM images, including diameter, length, and tilt angles. **(E)** CAD-based nanowire model constructed using the SEM-derived statistical parameters. **(F)** Three-dimensional FDTD simulation geometry illustrating the SEM-informed nanowire ensemble used for electromagnetic simulations.

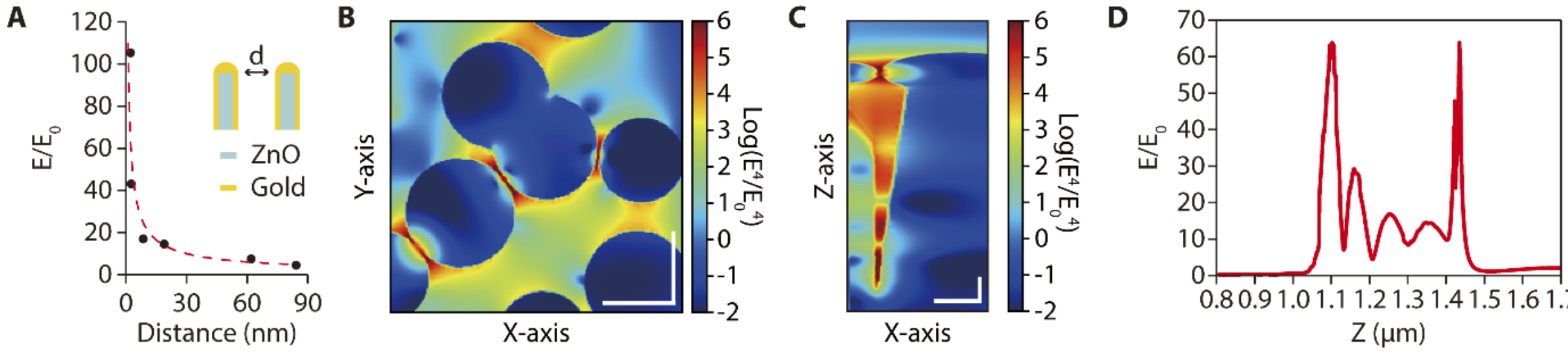

Fig. S5. FDTD simulation results of the AZ-SERS substrate. (A) Simulated enhancement factor as a function of distance from the nanowire surface. **(B)** XY-plane near-field enhancement map illustrating the lateral distribution of plasmonic electric field enhancement around neighboring nanowires. Scale bar, 50 nm. **(C)** XZ-plane electric field enhancement map showing strong enhancement localized near the nanowire surface along the vertical direction. Scale bar, 50 nm. **(D)** Line profile of the electric field distribution along the z-axis.

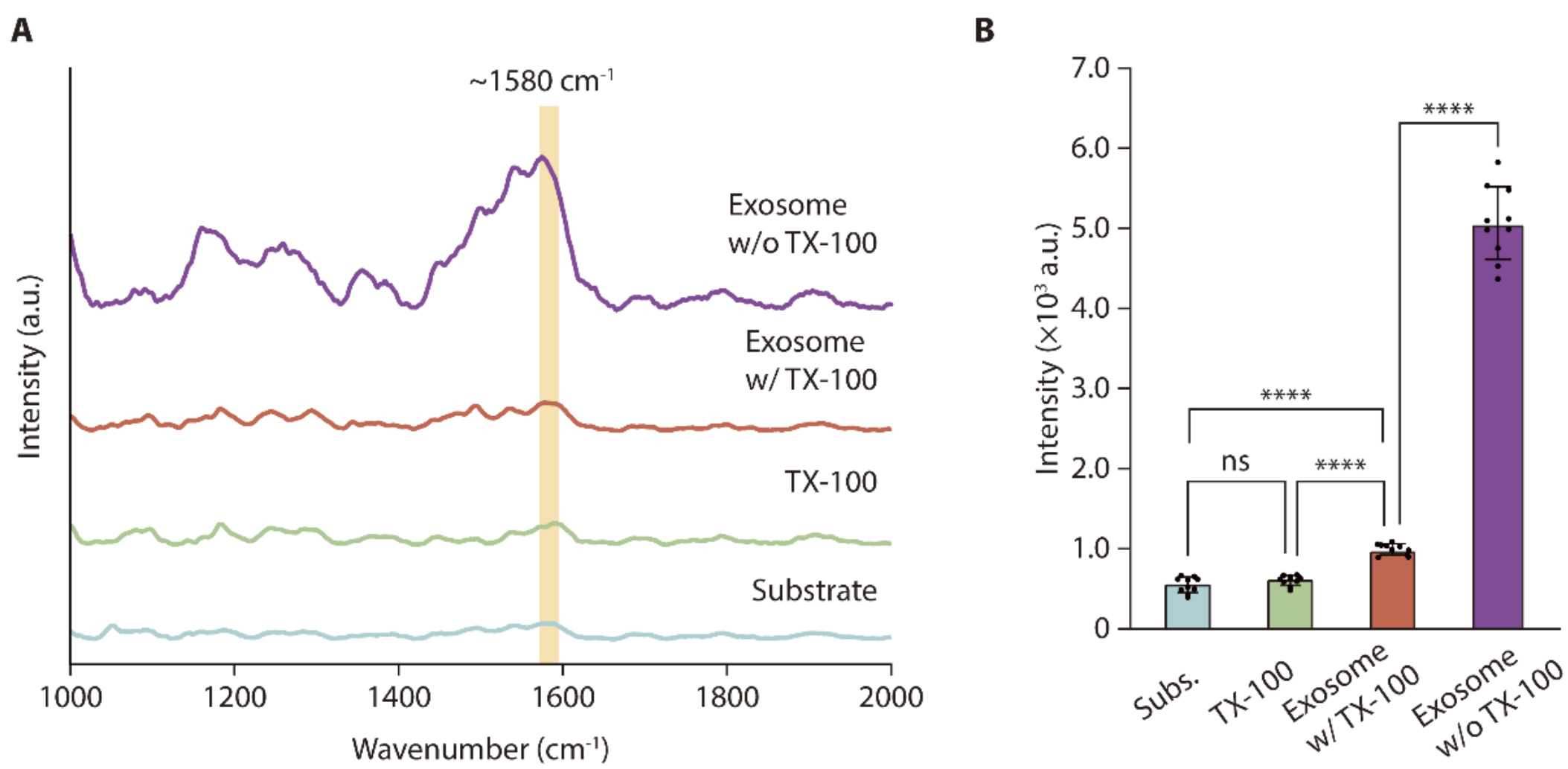

Fig. S6. Membrane-dependent SERS signal disruption by Triton X-100 (TX-100). (A) Representative SERS spectra of AZ-SERS substrates under four conditions: intact exosomes, exosomes treated with Triton X-100, Triton X-100 alone, and the bare substrate. **(B)** Quantitative comparison of SERS intensity at 1580 cm^{-1} under different conditions. Triton X-100 treatment results in a significant reduction of the exosome-associated SERS signal, indicating that the measured response depends on intact vesicular membranes. One-way ANOVA with the correction of Tukey's post hoc test. Bar graph data are presented as mean ± s.d. ****$p < 0.0001$.

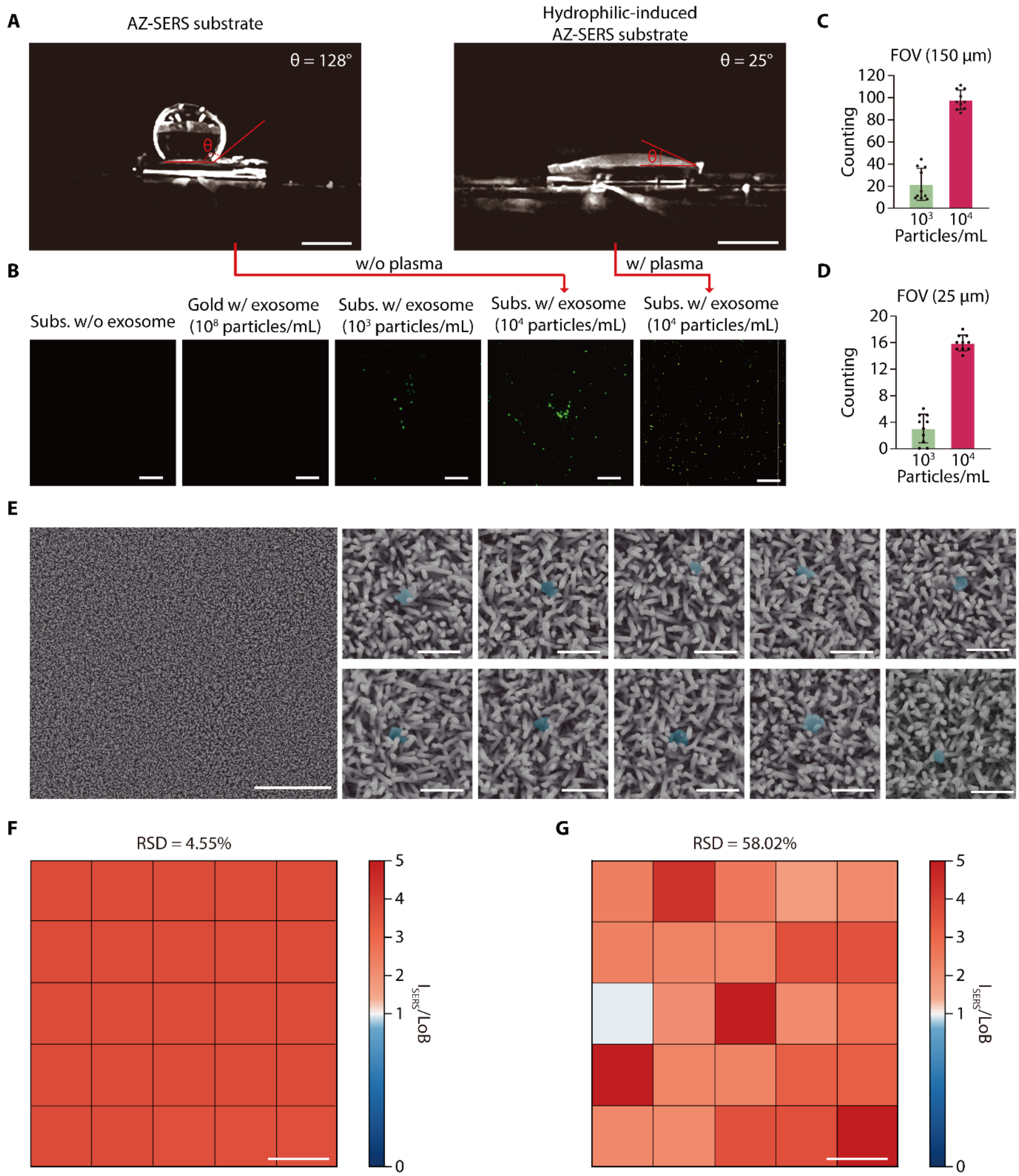

Fig. S7. Spatial distribution and occupancy of exosomes on AZ-SERS substrates at low concentrations. (A) Representative side-view images of droplets on AZ-SERS substrates with different surface wettabilities. The untreated AZ-SERS substrate exhibits a hydrophobic surface with a contact angle of 128°, whereas plasma treatment renders it hydrophilic, reducing the contact angle to 25°. Red lines indicate the measured contact angles. Scale bars, 1 mm. **(B)** Representative fluorescence images of AZ-SERS substrates without exosomes, gold substrates with exosomes, and AZ-SERS substrates with exosomes at 10^3 and 10^4 particles/mL. The

rightmost panel shows an AZ-SERS substrate subjected to plasma treatment under otherwise identical conditions. Exosomes are visualized with fluorescent imaging. Scale bars, 25 µm. **(C)** Quantification of exosome counts within a 150 × 150 µm² field of view (FOV) at 10^3 and 10^4 particles/mL, showing large variability at 10^3 particles/mL. Bar graph data are presented as mean ± s.d. **(D)** Exosome counts within the central ROI under the same conditions, demonstrating significantly higher and more reproducible occupancy at 10^4 particles/mL. Bar graph data are presented as mean ± s.d. **(E)** Representative SEM images acquired under identical conditions to the SERS measurements, confirming the presence of exosomes in the central region of the AZ-SERS substrate. Magnified images show approximately 10 exosomes within a 20 × 20 µm² area. Scale bars, 5 µm (left) and 500 nm (right). **(F)** ROI-based 5 × 5 SERS intensity heat map at 10^4 particles/mL, showing uniformly LoB-positive signals with low variability (RSD = 4.55%). Scale bars, 5 µm. **(G)** ROI-based 5 × 5 SERS intensity heat map at 10^3 particles/mL, showing LoB-positive but spatially heterogeneous signals with high variability (RSD = 58.02%). Scale bars, 5 µm.

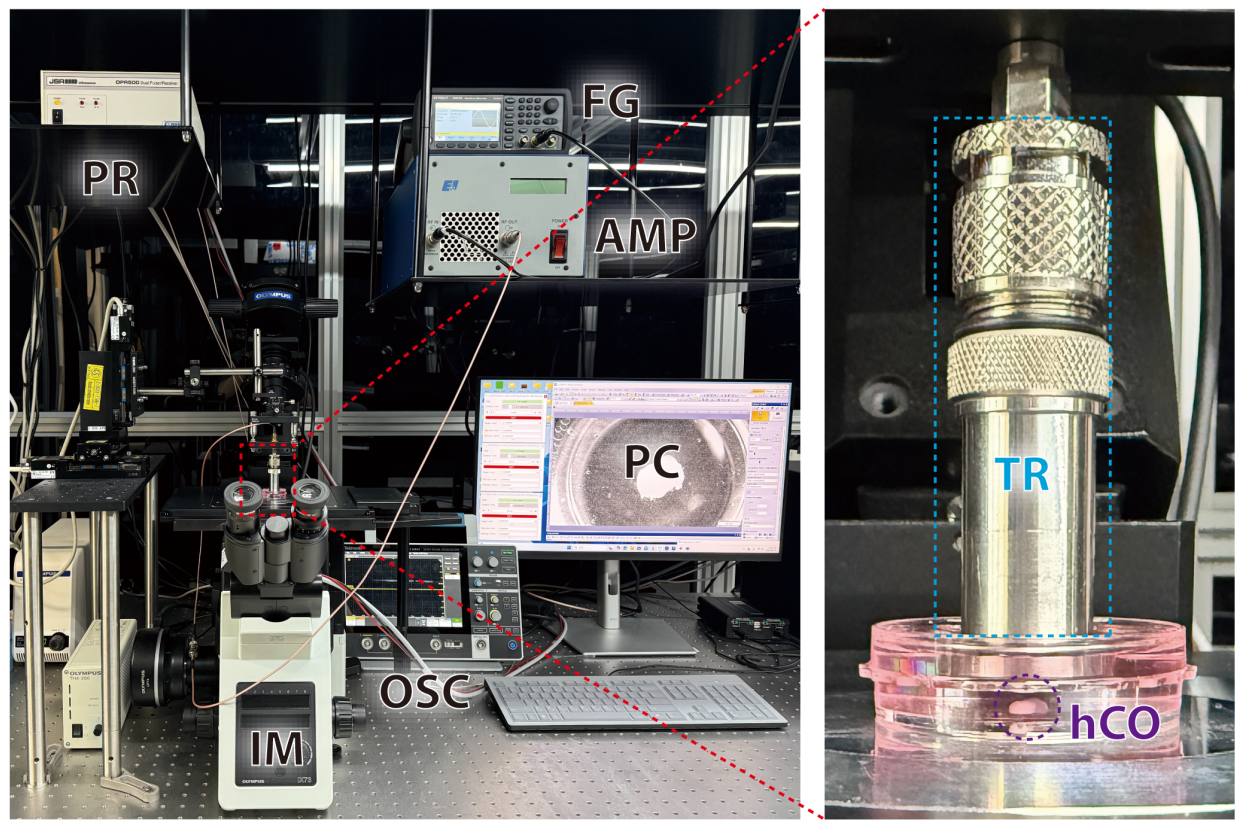

Fig. S8. Photograph of the experimental setup for LIPUS stimulation. PR, pulser receiver; FG, function generator; AMP, amplifier; OSC, oscilloscope; IM, inverted microscope; TR, transducer; PC, personal computer.

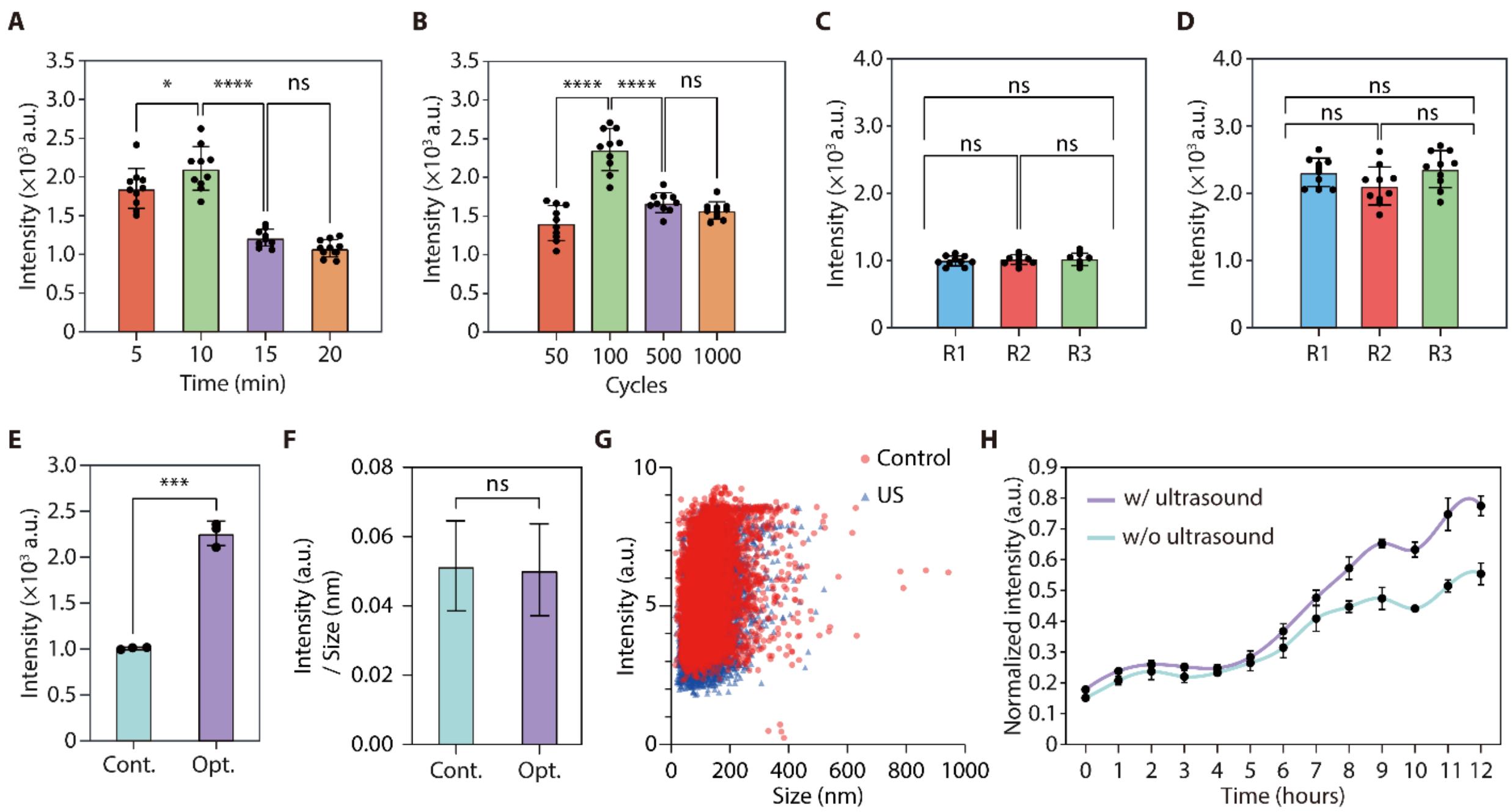

Fig. S9. Ultrasound parameter–dependent exosome secretion and release dynamics. Exosome-associated SERS intensity with varying ultrasound stimulation times **(A)** and cycle number **(B)**. **(C)** SERS intensity of exosomes under control conditions without ultrasound. R1–3 represent replicate measurements from three independent samples. **(D)** SERS intensity of exosomes under optimized stimulation. In **(A – D)**, one-way ANOVA with the correction of Tukey's post hoc test. **(E)** Quantification of exosome-associated SERS intensity under optimized conditions. **(F)** NTA results for size and intensity (n=5000). In **(E and F)**, a two-tailed, unpaired t-test was used. **(G)** NTA analysis of exosome size and intensity with or without ultrasound stimulation. NTA revealed no significant differences in size distribution and scattering intensity between exosomes from control (red) and ultrasound-stimulated (blue) conditions. **(H)** Time-course analysis following ultrasound exposure (n=5). Bar graph data are presented as mean ± s.d. *$p < 0.05$, ****$p < 0.0001$. Cont., control; Opt., optimized condition.

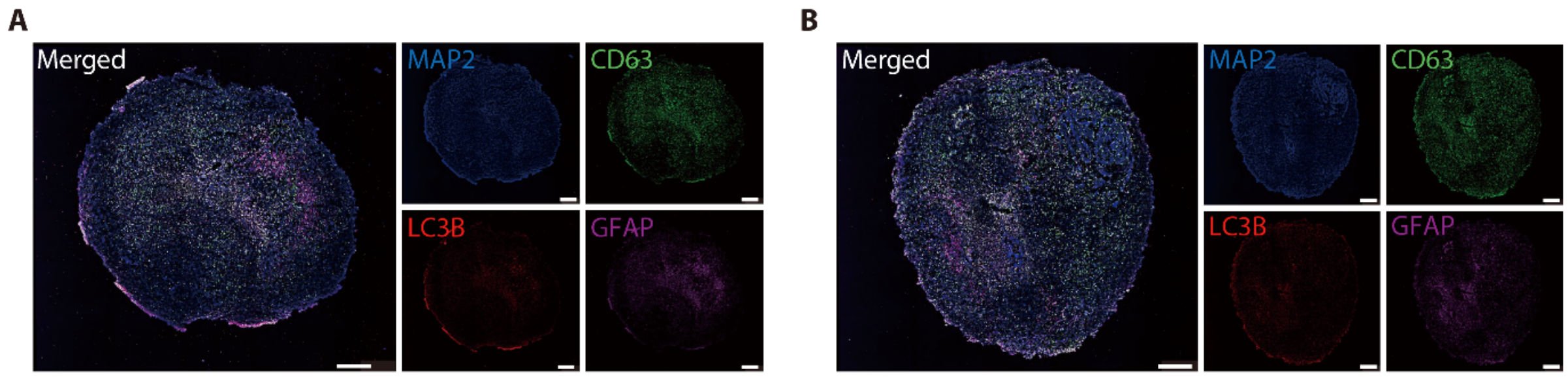

Fig. S10. Representative immunofluorescence images showing qualitative early post-stimulation changes in exosome release pathways following LIPUS stimulation. Organoids were stained for MAP2 (blue), CD63 (green), LC3B (red), and GFAP (magenta) and observed immediately after stimulation **(A)** and at 24 hours **(B)**. Scale bars, 200 μm.

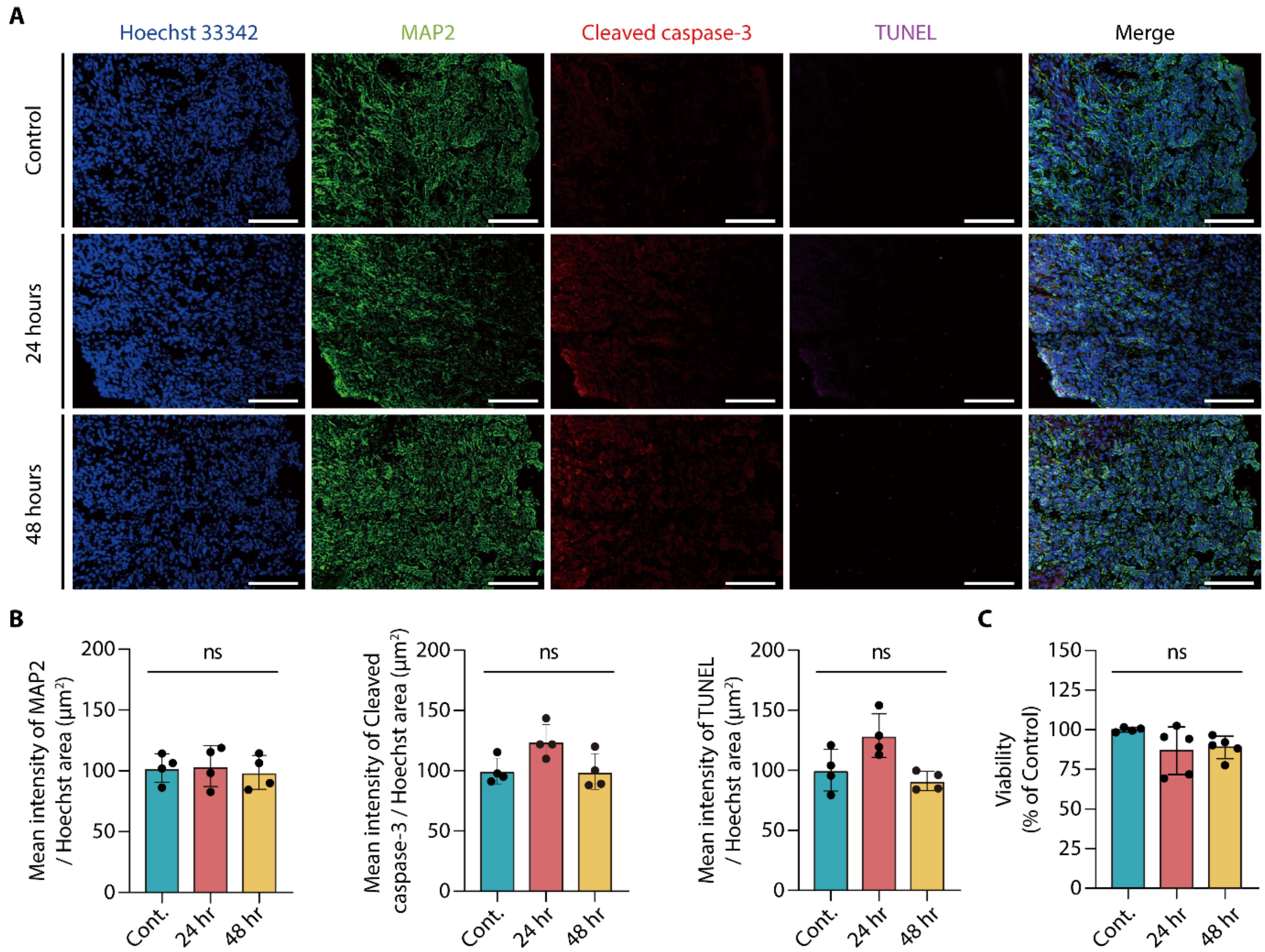

Fig. S11. Evaluation of neuronal marker expression, apoptosis-associated markers, and viability following LIPUS stimulation in hCO. (A) Representative immunofluorescence images of hCO sections stained for nuclei (Hoechst 33342), MAP2, Cleaved Caspase-3, and TUNEL in control organoids and at 24 h and 48 h after LIPUS stimulation under optimized conditions. Scale bars, 100 μm. **(B)** Quantification of normalized fluorescence intensities of MAP2, Cleaved Caspase-3, and TUNEL signals (normalized to Hoechst-stained area) in control, 24 h, and 48 h post-stimulation groups. **(C)** Cell viability assessed by MTT assay in control hCOs and at 24 h and 48 h after LIPUS stimulation, expressed as percentage of control. In **(B and C)**, one-way ANOVA with the correction of Dunnett's post hoc test was used. Bar graph data are presented as mean ± s.d.

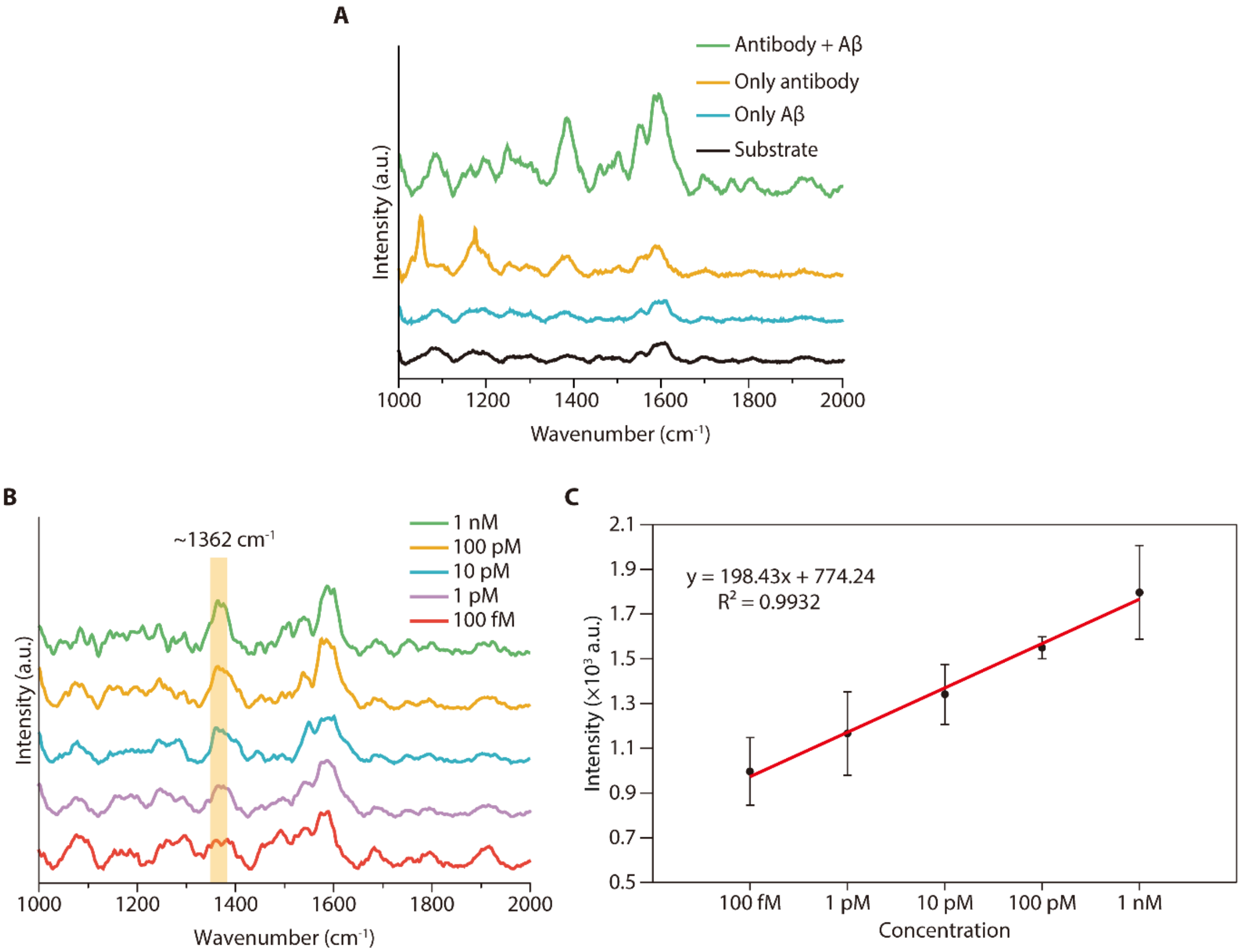

Fig. S12. SERS detection and validation of Aβ-associated signals. (A) Comparison of SERS spectra acquired from four conditions: antibody-functionalized substrates exposed to free Aβ, antibody-only controls, antibody-free substrates exposed to free Aβ, and the bare substrate; free Aβ was tested at a concentration of 1 nM. **(B)** Representative SERS spectra acquired from antibody-functionalized substrates incubated with Aβ, showing a reproducible spectral feature near 1362 cm^{-1}. **(C)** SERS intensity of the 1362 cm^{-1} band measured as a function of Aβ concentration, showing linear scaling.

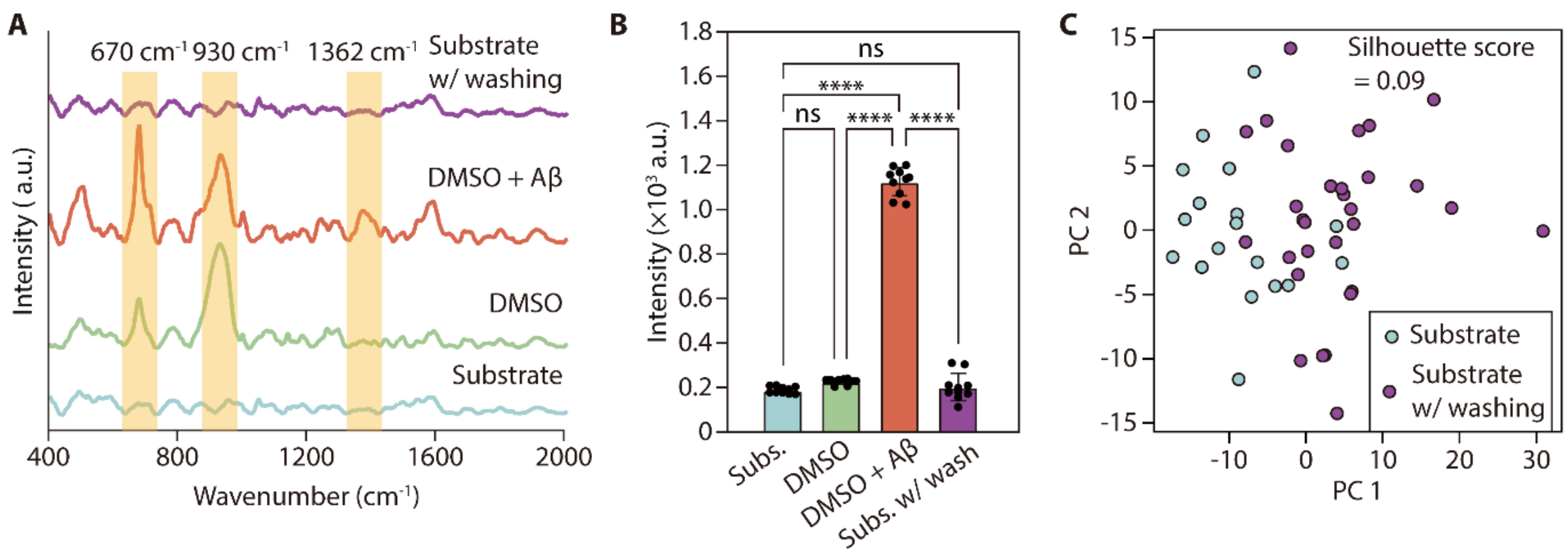

Fig. S13. Control experiments informing the interpretation of the 1362 cm⁻¹ signal. (A) SERS spectra of the substrate alone, DMSO-treated, DMSO + Aβ–treated, and DMSO + Aβ–treated samples followed by washing. The 1362 cm⁻¹ feature is observed under Aβ-treated conditions and is markedly reduced after washing. **(B)** Quantification of the SERS intensity at 1362 cm⁻¹ under each condition. One-way ANOVA with the correction of Tukey's post hoc test. Bar graph data are presented as mean ± s.d. ****$p < 0.0001$. **(C)** PCA comparison of spectra acquired from the substrate before and after washing, showing no meaningful separation.

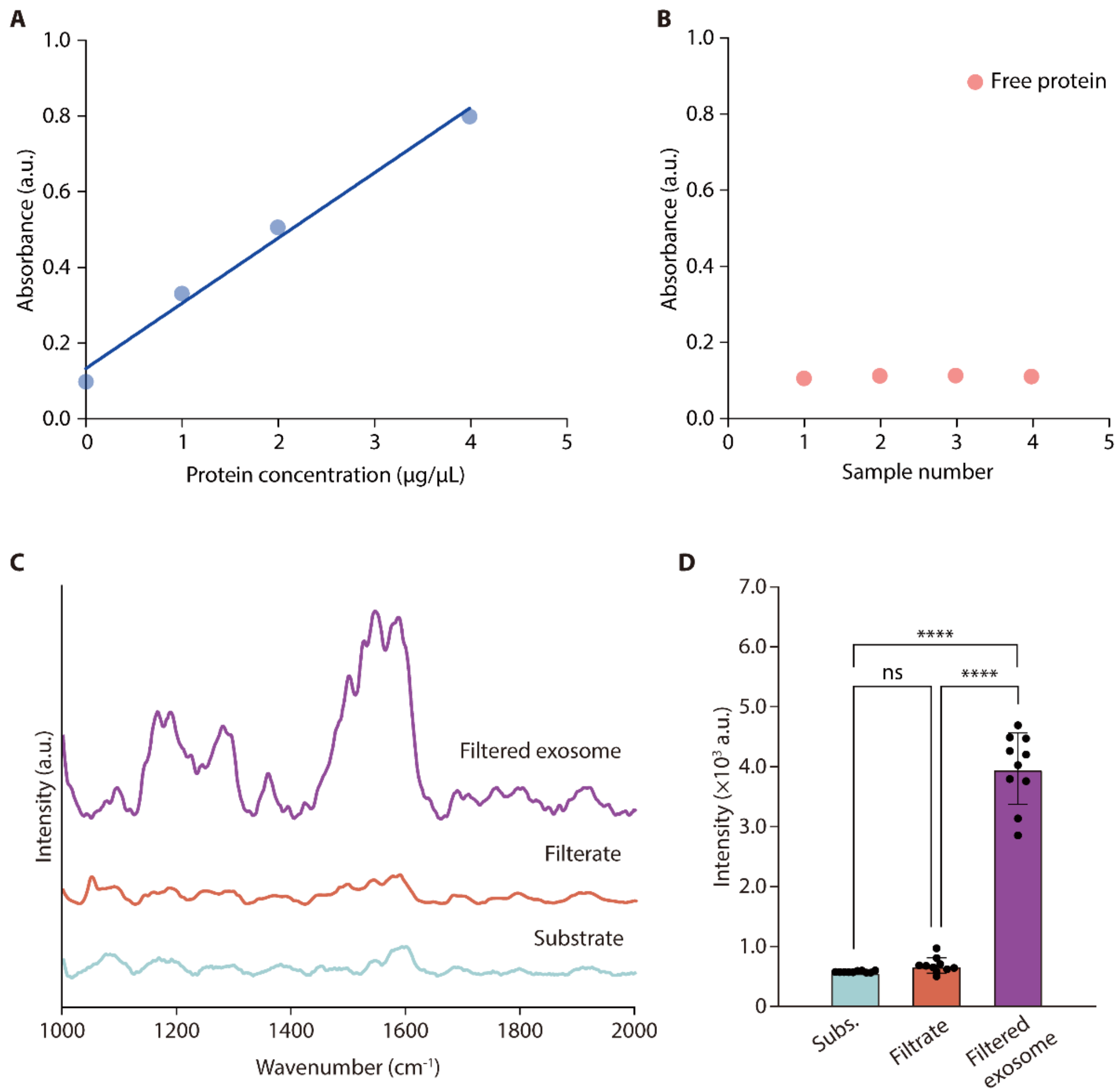

Fig. S14. Evaluation of residual free protein contribution to exosome SERS signals. (A) BCA standard curve used for protein quantification. **(B)** BCA assay results for the filtrate obtained after passing the purified exosome solution through a 100 kDa molecular-weight cutoff filter, showing absorbance levels below the detection limit compared to protein standards. **(C)** Representative SERS spectra acquired from the filtered exosome fraction, the filtrate (<100 kDa), and the bare substrate. **(D)** Quantitative comparison of SERS intensity at 1580 cm^{-1} under the indicated conditions. The filtered exosome fraction exhibits a strong SERS signal, whereas the filtrate shows signal levels comparable to the bare substrate. One-way ANOVA with the correction of Tukey's post hoc test. Bar graph data are presented as mean ± s.d. ****$p < 0.0001$.

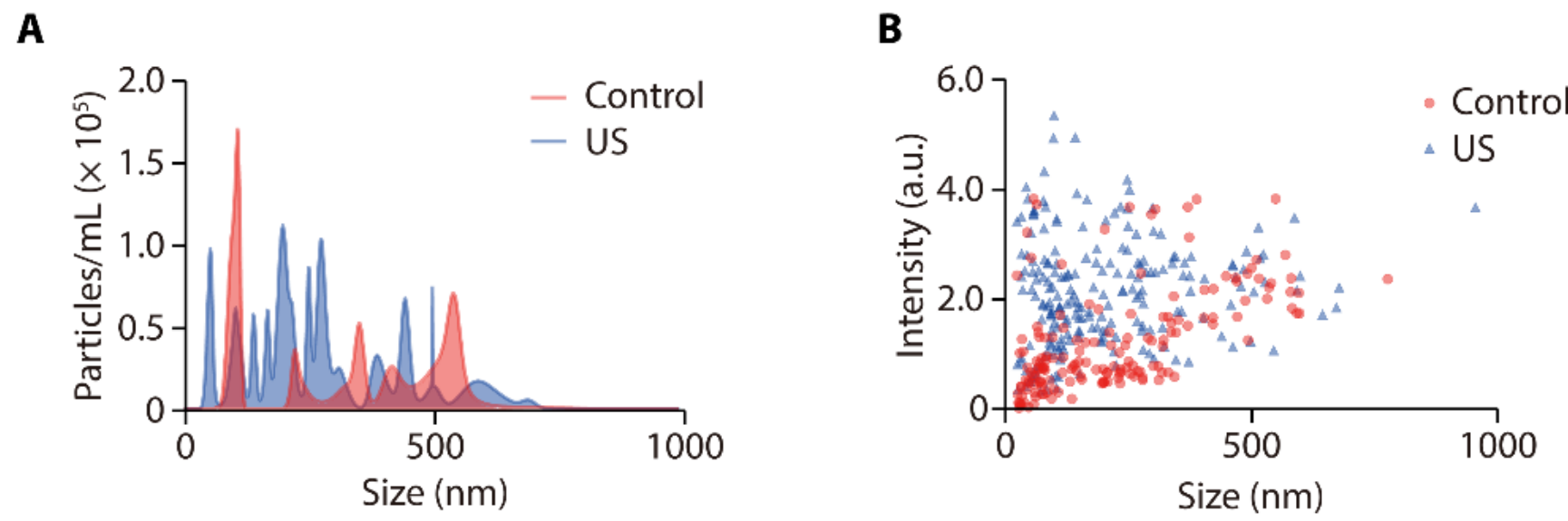

Fig. S15. Fluorescence NTA analysis of FITC-labeled Aβ-containing exosomes. (A) Size distribution of exosomes isolated from Aβ-treated hCOs with (blue) or without (red) ultrasound stimulation. **(B)** Scatter plots of particle-by-particle fluorescence intensity and size.

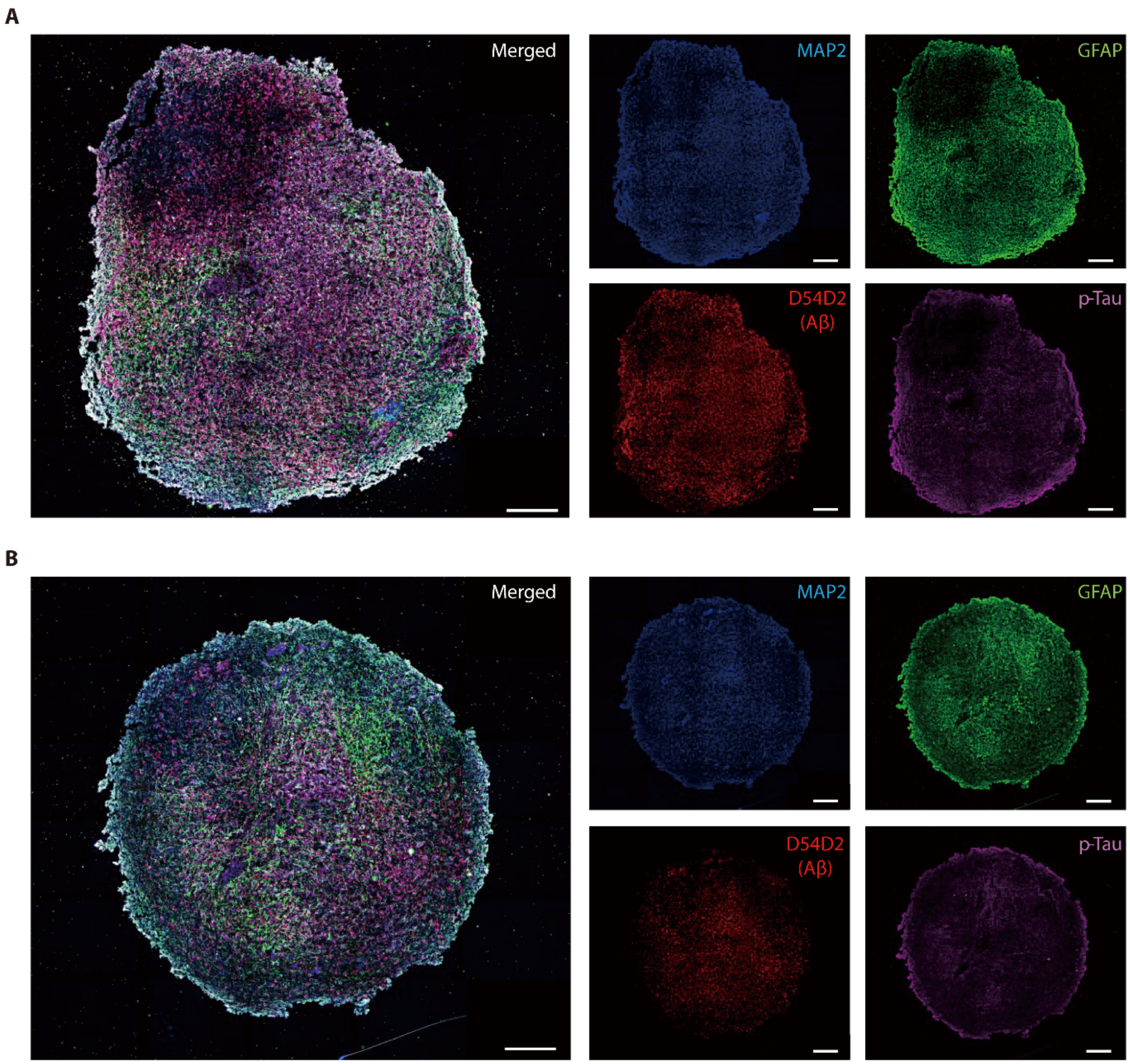

Fig. S16. Representative immunofluorescence images with and without LIPUS stimulation. **(A)** Control (no LIPUS). **(B)** With LIPUS stimulation. Organoids were stained for MAP2 (blue), GFAP (green), D54D2 (red), and p-Tau (magenta). Scale bars, 200 µm.

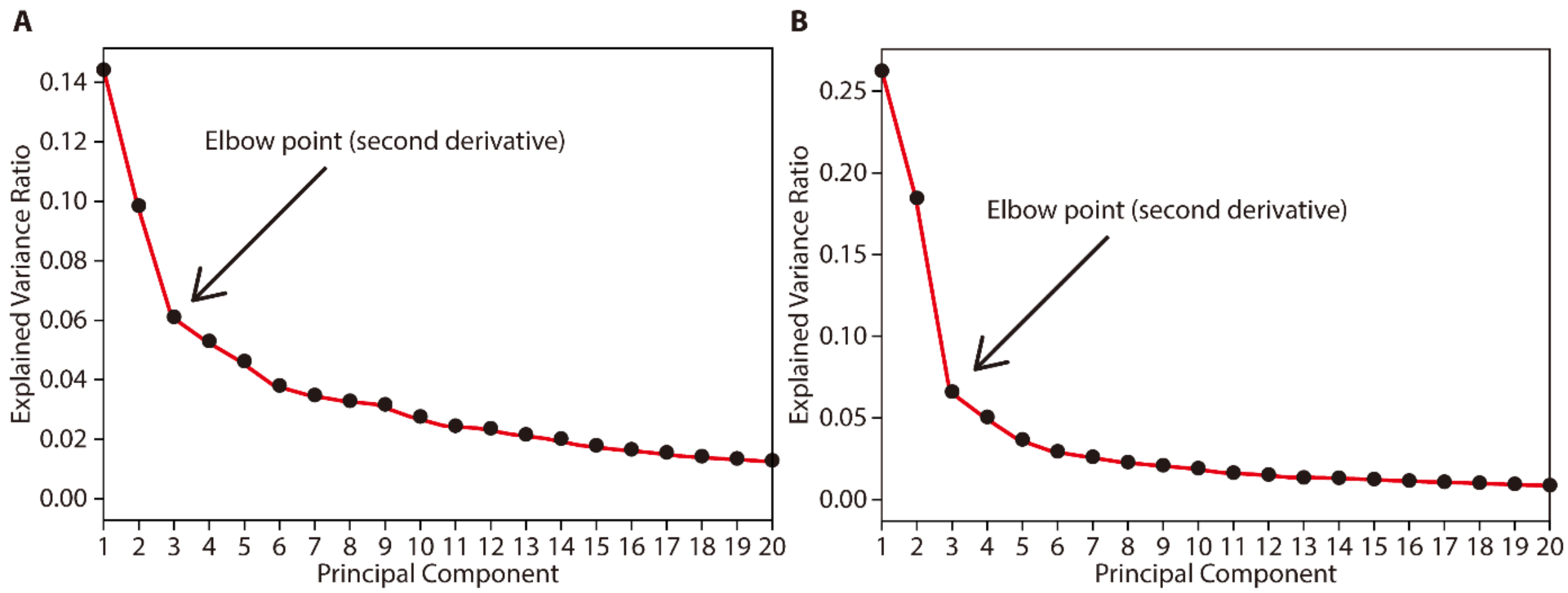

Fig. S17. Elbow analysis to select the number of principal components in PCA space. (A) Without ultrasound. **(B)** With ultrasound. Black points indicate the explained variance ratio for each principal component and the red line shows the decay of explained variance ratio as the principal component for PCA increases. The elbow point, indicating the optimal number of principal components, was yielded by second-derivative analysis, which showed that three principal components were the most appropriate for PCA analysis in Figs. 4I, J.

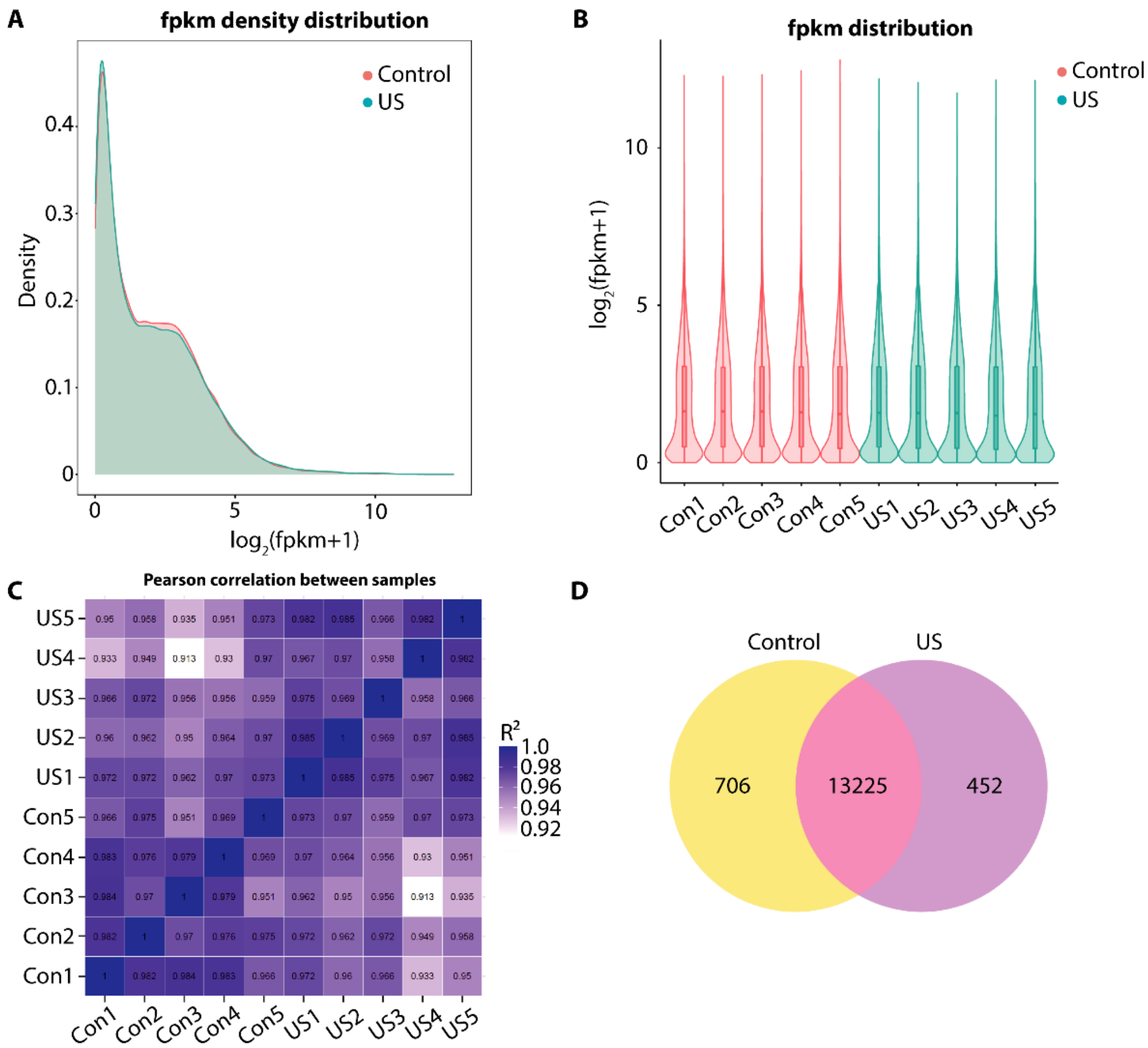

Fig. S18. Quality check for RNA sequencing data. (A) FPKM density distribution plot for both control group and ultrasound-treated group. **(B)** FPKM distribution for each organoid sample. **(C)** Pearson correlation between organoid samples. **(D)** The number of expressed genes for both control and ultrasound treated group.

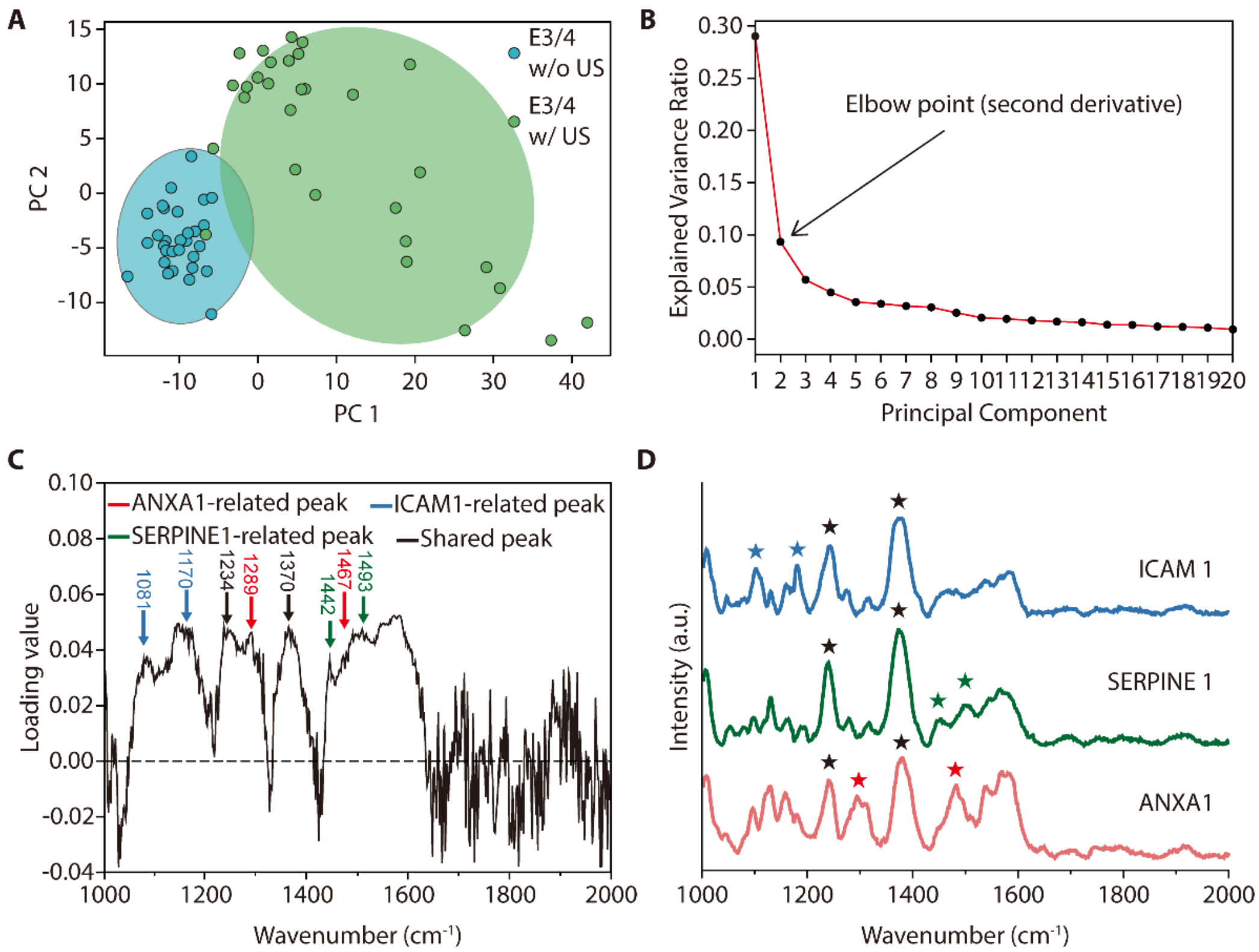

Fig. S19. Ultrasound-induced changes in exosome SERS fingerprints and their relationship to AD-associated exosomal proteins. (A) PCA of exosome-derived SERS spectra acquired from APOE E3/E4 organoids with and without ultrasound stimulation, showing clear separation driven by ultrasound application (silhouette score = 0.51). **(B)** Explained variance ratio as a function of principal component number. The elbow point was determined by second-derivative analysis. **(C)** Loading plot of the principal components, highlighting Raman bands that dominantly contribute to PCA separation. Colored markers indicate Raman bands overlapping with reference spectra of ICAM1 (blue), SERPINE1 (green), and ANXA1 (red), while black markers denote shared peaks among these proteins. **(D)** Representative SERS spectra of ICAM1, SERPINE1, and ANXA1 measured on the AZ-SERS substrate without washing. Asterisks indicate dominant Raman bands that overlap with PCA-driving spectral features observed in exosome-derived measurements.

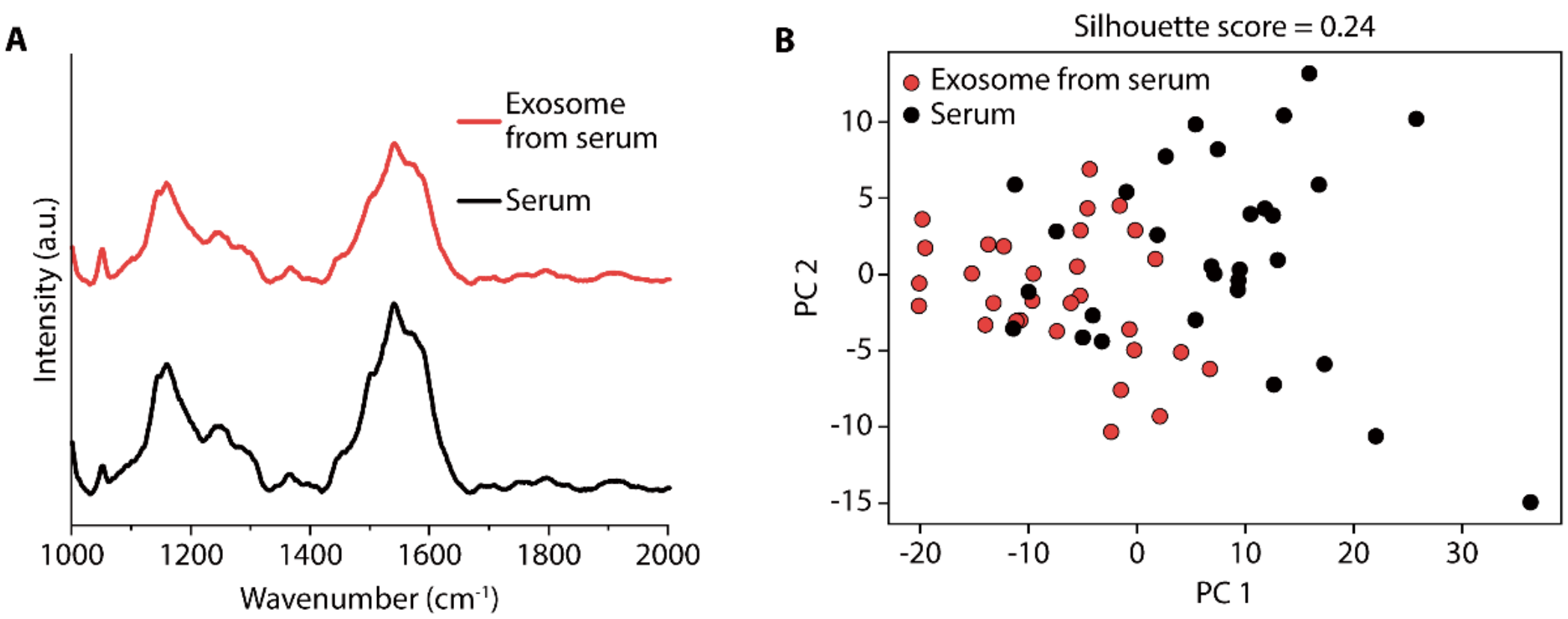

Fig. S20. Evaluation of exosome-derived SERS fingerprints in a complex serum matrix. (A) Representative SERS spectra acquired from purified exosomes isolated from serum (red) and from serum alone (black) under identical measurement conditions. **(B)** PCA of SERS spectra from serum-derived exosomes and serum samples. The low silhouette score (0.24) indicates no clear separation between the two groups, suggesting that non-specific serum components do not dominate or distort the PCA-based classification.

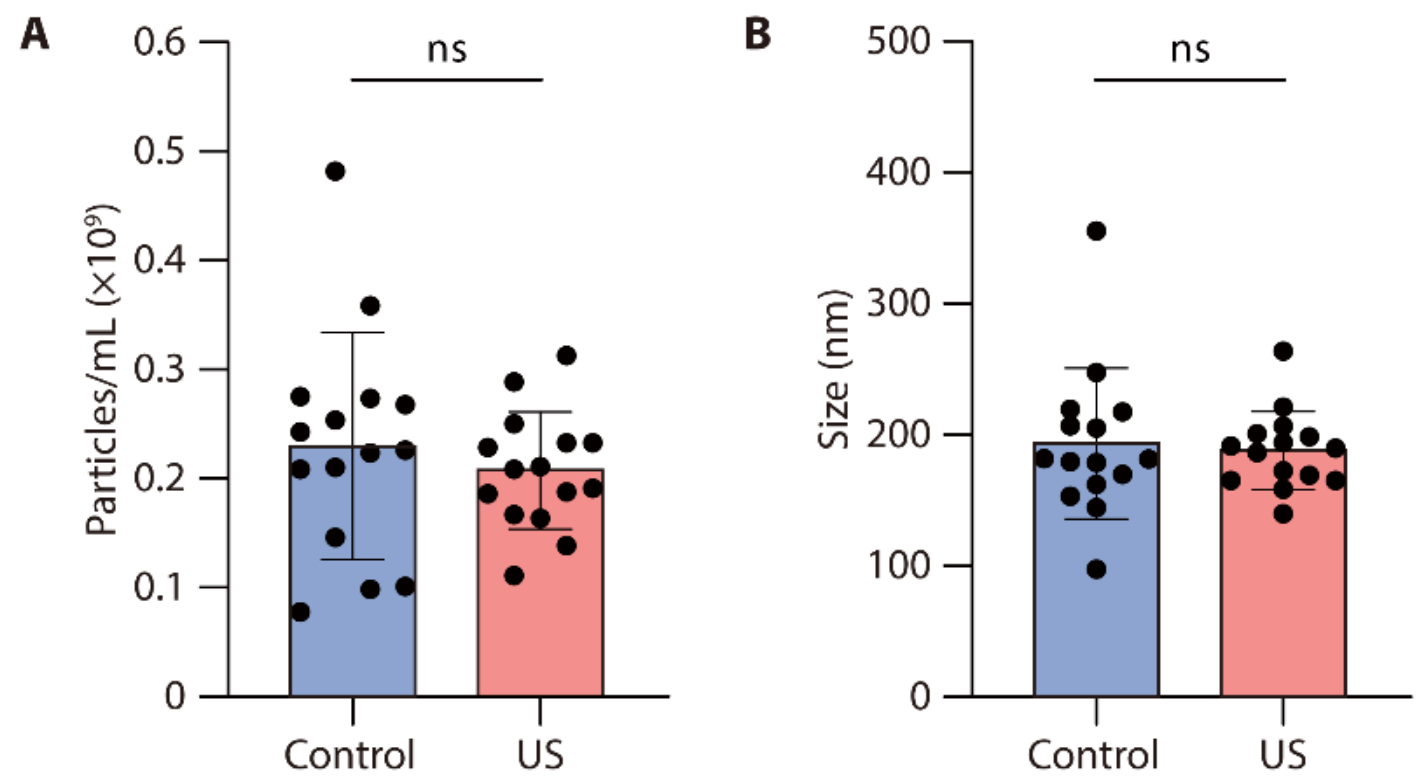

Fig. S21. LIPUS response in 2D-cultured U87 cells. (A) Exosome concentration measured by NTA in 2D-cultured U87 cells with and without LIPUS stimulation using the acoustic parameters optimized for hCOs (70 kPa, 10 min, 100 cycles). No significant difference was observed between groups. **(B)** Particle size distribution of exosomes released from U87 cells with and without LIPUS stimulation, showing no detectable change in vesicle size. A two-tailed, unpaired t-test was used. Bar graph data are presented as mean ± s.d.

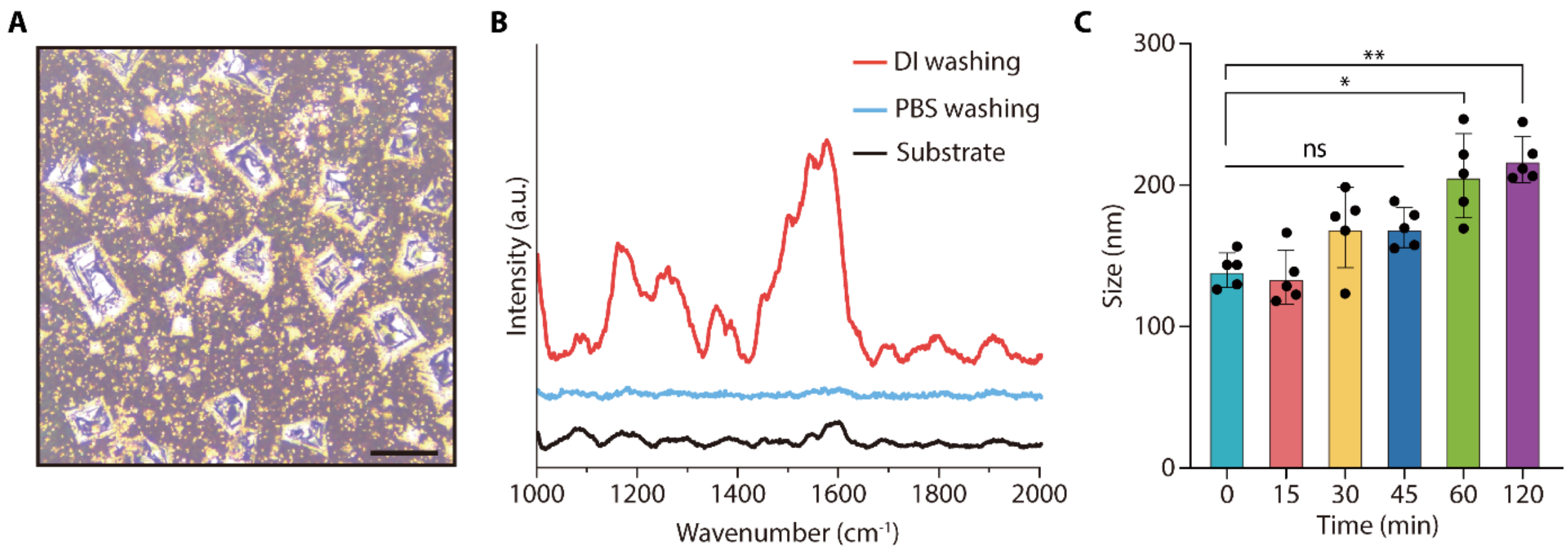

Fig. S22. Validation of DI water washing. (A) Representative optical image of the AZ-SERS substrate after PBS washing. Scale bars, 50 µm. **(B)** Comparison of SERS spectra acquired after DI water washing, PBS washing, and from the bare substrate. PBS washing failed to produce measurable SERS signals, in contrast to DI water washing, which enabled reproducible spectral acquisition. **(C)** Time-dependent changes in exosome size during incubation in DI water measured by NTA. Exosome size remained stable for up to approximately 45 min, followed by a gradual increase at longer incubation times. One-way ANOVA with the correction of Dunnett's post hoc test. Bar graph data are presented as mean ± s.d. $^{**}p < 0.01$, $^{*}p < 0.05$.

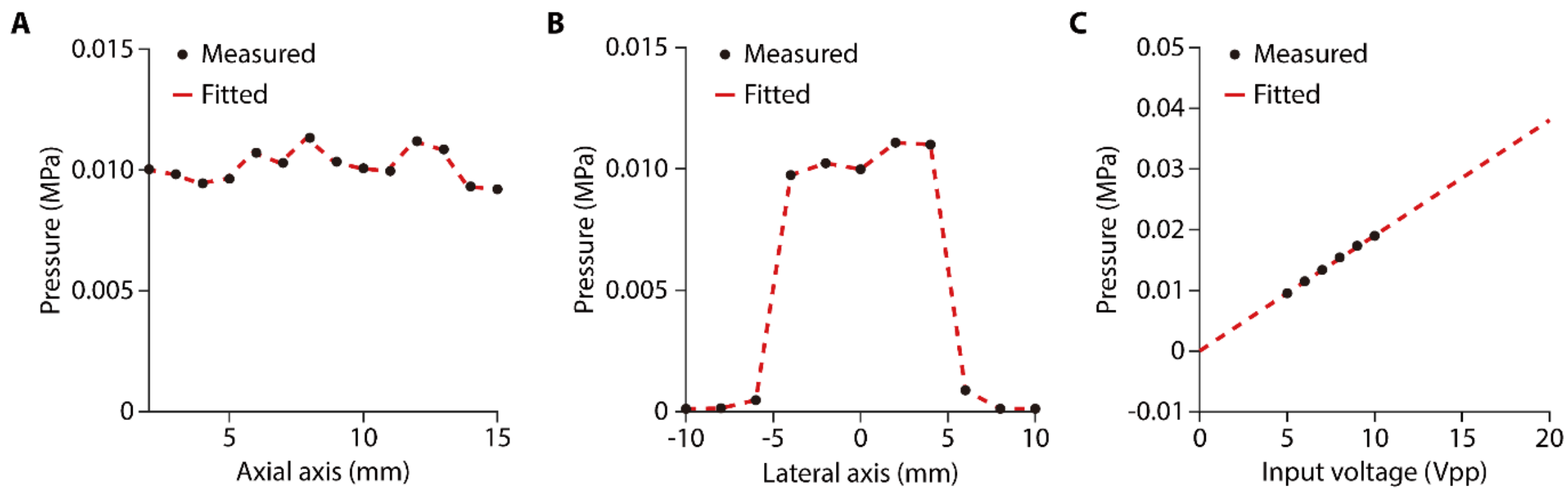

Fig. S23. Acoustic characterization of the ultrasound transducer. (A) Axial pressure profile measured and fitted. **(B)** Lateral pressure profile measured and fitted. **(C)** Linear relationship between input voltage and measured pressure.

Table S1. Protein information used for reference SERS measurements.

Protein	Cat. No	Supplier	Reconstitution solvent	Reconstitution concentration
Annexin A1/ANXA1	HY-P7512	MedChemExpress	ddH2O	100 μg/mL
Serpin E1	HY-P71077	MedChemExpress	ddH2O	100 μg/mL
ICAM-1/CD54	HY-P72621	MedChemExpress	ddH2O	100 μg/mL

Table S2. Antibody information applied in immunohistochemistry (IHC) and western blotting (WB).

Antibody	Cat. No	Supplier	Clonality/isotype	Dilution	Host	Method	RRID
Anti-TSG101	ab83	Abcam	monoclonal/IgG	1:200 (IHC) 1:500 (WB)	mouse	WB, IHC	AB_306450
Anti-Cleaved caspase-3	NB100-56113	Novus Biologicals	polyclonal/IgG	1:1000 (WB)	rabbit	WB	AB_3073989
Anti-LC3B	2775	Cell Signaling Technology	polyclonal/IgG	1:200 (IHC) 1:1000 (WB)	rabbit	WB, IHC	AB_915950
Anti-β-actin	ab6276	Abcam	monoclonal/IgG1	1:1000 (WB)	mouse	WB	AB_2223210
Anti-Amyloid beta (Aβ, D54D2)	8243T	Cell Signaling Technology	monoclonal	1:200 (IHC)	rabbit	IHC	AB_2797642
Anti-MAP2	ab5392	Abcam	polyclonal/IgY	1:200 (IHC)	Chicken	IHC	AB_2138153
Anti-GFAP	13-0300	Invitrogen	monoclonal/IgG2a	1:200 (IHC)	rat	IHC	AB_2532994
Anti-Thr181 (p-Tau)	MN1050	Invitrogen	monoclonal/IgG1	1:200 (IHC)	mouse	IHC	AB_223651
